# Integrase-Deficient Lentiviral Vector as a Platform for Efficient CRISPR/Cas9-Mediated Gene Editing for Mucopolysaccharidosis IVA

**DOI:** 10.3390/ijms26146616

**Published:** 2025-07-10

**Authors:** Fnu Nidhi, Shunji Tomatsu

**Affiliations:** 1 Nemours Children’s Health, Wilmington, DE 19803, USA; fnu.nidhi@nemours.org; 2 Faculty of Arts and Sciences, University of Delaware, Newark, DE 19716, USA; 3 Department of Pediatrics, Graduate School of Medicine, Gifu University, Gifu 501-1193, Japan; 4 Department of Pediatrics, Thomas Jefferson University, Philadelphia, PA 19107, USA

**Keywords:** IDLV, CRISPR/Cas9, MPS IVA, gene editing

## Abstract

Mucopolysaccharidosis IVA (MPS IVA) is a lysosomal storage disorder causing systemic skeletal dysplasia due to a deficiency of N-acetyl-galactosamine-6-sulfate sulfatase (GALNS) enzyme activity, leading to the impaired degradation and accumulation of glycosaminoglycans (GAGs), keratan sulfate (KS) and chondroitin-6-sulfate. While treatments such as enzyme replacement therapy (ERT) and hematopoietic stem cell transplantation (HSCT) are available, they have significant limitations regarding efficacy in skeletal tissues and long-term safety, highlighting the need for more effective therapies. We evaluated a novel gene therapy approach using a dual Integrase-deficient lentiviral vector (IDLV) to deliver an expression cassette that includes human *GALNS* cDNA and Cas9 sgRNA, targeting the upstream region of the mouse *Galns* initial codon. This approach leverages the endogenous promoter to drive transgene expression. We assessed in vitro transduction, editing, and functional correction in NIH3T3 and MPS IVA mouse fibroblasts. In vivo efficacy was successfully evaluated via the facial vein injection in MPS IVA newborn mice. In vitro, this IDLV platform demonstrated supraphysiological GALNS activity in cell lysate, resulting in the normalization of KS levels. In vivo direct IDLV platform in newborn MPS IVA mice led to sustained plasma GALNS activity, reduced plasma KS, and favorable biodistribution. Partial correction of heart and bone pathology was observed, with no vector toxicity and minimal antibody responses. This dual IDLV-CRISPR/Cas9 approach effectively mediated targeted *GALNS* knock-in, yielding sustained enzyme activity, reduced KS storage, and partial pathological amelioration in MPS IVA mice. In conclusion, IDLVs represent an efficient, safe platform for delivering the CRISPR/Cas9 gene editing system for MPS IVA.

## 1. Introduction

Mucopolysaccharidosis IVA (MPS IVA) or Morquio A syndrome (ORPHA: 309297, OMIM: 253000) is a rare, autosomal recessive lysosomal storage disorder (LSD) caused by mutations in the *GALNS* gene, resulting in the deficiency of N-acetyl-galactosamine-6-sulfate sulfatase (GALNS) enzyme [1,2,3,4,5]. The deficiency of this enzyme results in impaired degradation of specific glycosaminoglycans (GAGs), keratan sulfate (KS) and chondroitin 6-sulfate (C6S), leading to their accumulation in lysosomes [2,3,6,7]. The accumulation of these GAGs leads to bone growth impairment by interfering with chondrocyte homeostasis, increasing matrix-degrading enzymes that contribute to cartilage matrix breakdown, and ultimately leading to cell apoptosis [8,9,10]. This further results in cartilage imbalance, defective ossification, and reduced bone mineral density (BMD), contributing to severe skeletal dysplasia features, including prominent forehead, abnormal facial structure, short-trunk dwarfism, cervical spine instability, kyphoscoliosis, hip dysplasia, genu valgum, hypermobile joints, and waddling gait, with additional narrowing of the trachea, restrictive lung disease, and spinal cord compression and respiratory failure as the primary cause of death [1,8,9,11,12].

The current standard treatment for MPS IVA is enzyme replacement therapy (ERT), where a recombinant enzyme is infused weekly in patients costing USD 500,000/year/25 kg (USA), with limited impact on skeletal dysplasia, requirement of additional surgical intervention, and immune response against the enzyme posing severe limitations to be addressed [13,14,15,16,17,18]. Another standard therapy employed is allogeneic hematopoietic stem cell transplantation (HSCT), where stem cells from a healthy donor are transplanted into the patients, where some improvement has been reported in BMD, pulmonary function, and activity of daily living in individual cases [15]. However, HSCT carries substantial risks, including graft-versus-host disease (GVHD), and its overall impact on bone deformities is limited. Successful outcomes depend on donor matching and institutional expertise, and the long-term efficacy and safety profile for MPS IVA remain unclear [15,19].

Gene therapy using classical and advanced CRISPR/Cas9 gene editing systems has garnered immense interest, as it focuses on correcting the underlying cause of the issue rather than addressing the heterologous spectrum of mutations that cause the disease [5]. CRISPR/Cas9 gene editing enables precise correction of genetic defects and has shown promise for LSDs like MPS, including MPS IVA. Recent studies have demonstrated successful knock-in of therapeutic genes (such as *GALNS*) into safe harbor loci, restoring enzyme activity and correcting disease phenotypes in vitro and in animal models [20,21]. The primary challenge lies in selecting a delivery vehicle that efficiently carries the CRISPR/Cas9 system. Most popular delivery vehicles are (1) adeno-associated virus (AAV) vector, with limited payload capacity, potential immunogenicity, and challenges with repeated dosing due to immune responses [22,23,24], and (2) lentiviral vectors (LVs) carrying larger genes but integrating randomly into the genome, raising concerns about insertional mutagenesis and oncogenesis [25,26]. With these limitations, a system is required to demonstrate episomal properties like AAV and high transduction efficiency like LVs.

Integrase-defective lentiviral vectors (IDLVs) provide a safer alternative by delivering CRISPR/Cas9 components without permanent integration, thereby reducing the risk of insertional mutagenesis associated with conventional LVs [27]. Unlike AAV, IDLVs can accommodate larger genetic payloads and avoid pre-existing immunity issues, while providing transient expression sufficient for gene editing and minimizing long-term off-target effects. IDLVs also allow for more controlled and potentially repeated dosing compared to AAV, and their non-integrating nature addresses major safety concerns associated with both AAV and LV systems [25,26].

In this study, we have leveraged IDLVs as a delivery platform for CRISPR/Cas9 to advance safe, efficient, and durable gene therapy for MPS IVA, potentially overcoming the barriers which current viral and non-viral systems face.

## 2. Results

A. In vitro validation of the IDLV-mediated CRISPR/Cas9 gene editing strategy.

### 2.1. IDLV-Mediated CRISPR/Cas9 Gene Editing Strategy Enhances the GALNS Gene Expression Through Targeted and Episomal Effects

**Figure 1:**
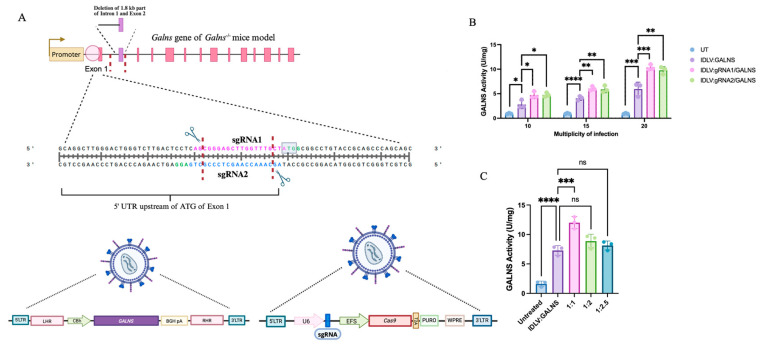
IDLV-mediated CRISPR/Cas9 gene editing strategy. (**A**) Schematic of the gene-editing approach. (**Top**) The genomic target is the 5′ untranslated region (UTR) of the mouse *Galns* gene, located upstream of the start codon (ATG) in Exon 1. The diagram depicts the specific *Galns*^-/-^ mouse model used, which contains a deletion in the gene. Two single guide RNAs, sgRNA1 and sgRNA2, are designed to create a double-strand break at the target site. The Protospacer Adjacent Motif (PAM) sequences are highlighted. (**Bottom**) A two-vector integrase-deficient lentivirus (IDLV) system is used to deliver the editing components. The donor template vector (**left**) contains the human *GALNS* cDNA driven by an Elongation Factor-1 alpha Short (EFS) promoter and followed by a Woodchuck Hepatitis Virus Posttranscriptional Regulatory Element (WPRE). This therapeutic cassette is flanked by 5′ and 3′ homology regions (HR) to facilitate integration. The CRISPR/Cas9 vector (**right**) uses a U6 promoter to express the sgRNA and an EFS promoter to express the Cas9 nuclease. This figure was created with Biorender.com. (**B**) Optimization of IDLV multiplicity of infection (MOI) in NIH3T3 fibroblasts. Data represent means ± SD, *n* = 3. (**C**) Optimization of IDLV transduction ratios (donor: CRISPR components). Data represent means ± SD, *n* = 3. * *p* < 0.05, ** *p* < 0.01, *** *p* < 0.001, **** *p* < 0.0001.

We employed an IDLV-mediated CRISPR/Cas9 system to target the 5′ untranslated region (UTR) just before the initial ATG codon of murine *Galns* exon 1 in our MPS IVA *Galns^-^*^/*-*^ mouse model, aiming to integrate the human *GALNS* cDNA. The system drove the endogenous *Galns* promoter, which expressed the human *GALNS* gene. Two sgRNAs were designed to induce targeted double-stranded breaks (DSBs) at distinct loci within the region of interest (ENSMUSG00000015027; GRCm39) (Figure 1A). The IDLV vectors carried either the expression cassette alone (IDLV:GALNS) or coupled with CRISPR/Cas9 components (IDLV:gRNA1/GALNS, IDLV:gRNA2/GALNS) (Figure 1A). The homologous repair template consisted of homology arms flanking the *GALNS* cDNA, leveraging the endogenous promoter activity upon successful homology-directed repair (HDR) integration and providing potential additive effects from episomal expression (Supplementary Figure S1).

Initially, we optimized the multiplicity of infection (MOI) and transduction ratio in NIH3T3 fibroblasts (Figure 1B). Significant dose-dependent increases in intracellular GALNS enzyme activity were observed at MOIs of 10, 15, and 20. The highest GALNS activity was achieved at an MOI of 20, with IDLV:gRNA1/GALNS and IDLV:gRNA2/GALNS groups showing 10.34 ± 0.57 U/mg and 9.77 ± 0.74 U/mg, respectively, significantly greater than untreated (UT) NIH3T3 fibroblasts (*p* < 0.0001; Figure 1C). Similarly, optimization of transduction ratios between IDLV carrying the transgene and CRISPR components showed the highest GALNS enzyme activity at a 1:1 ratio, yielding 12.46 ± 1.02 U/mg, demonstrating optimal balance for functional genomic integration and expression (Figure 1C).

These comprehensive analyses validated our dual-IDLV CRISPR/Cas9 system for targeted gene editing and stable GALNS expression, highlighting the importance of precise vector optimization. The system also demonstrated additive effects from episomal and genome-editing gene expression.

### 2.2. Quantification of Live-Cell EGFP Expression Reveals Episomal and HDR-Mediated GALNS Expression in IDLV-Transduced Fibroblasts

**Figure 2:**
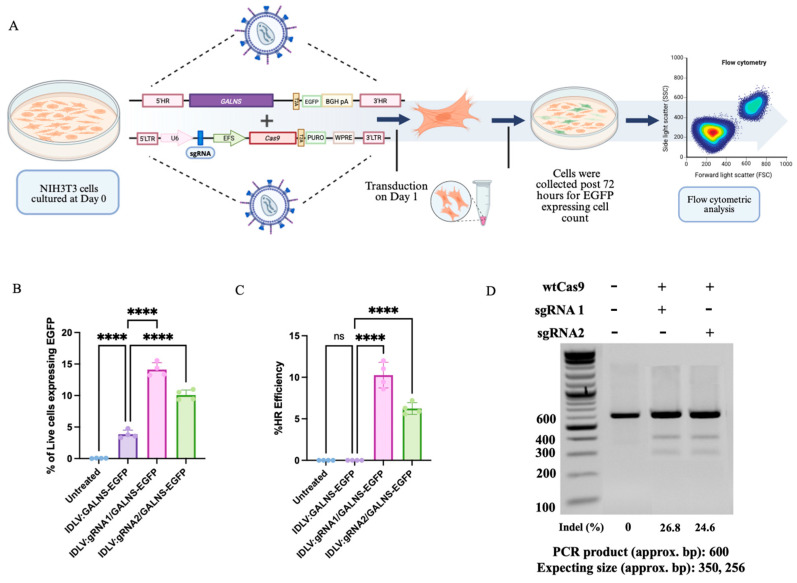
Evaluation of homologous recombination and CRISPR/Cas9 editing efficiency in NIH3T3 fibroblasts. (**A**) Schematic experimental approach for assessing HDR-mediated gene editing. NIH3T3 cells were cultured on day 0 and transduced on day 1 with IDLV vectors delivering either the donor-only cassette (*GALNS*-*egfp*) or HDR-competent constructs (IDLV:gRNA1:GALNS and IDLV:gRNA2:GALNS). Cells and media were collected post-72 h for flow cytometric analysis. This figure was created with Biorender.com. (**B**) Percentage of live NIH3T3 cells expressing EGFP post-transduction with donor-only and HDR-competent vectors (*n* = 4). (**C**) Quantification of homologous recombination efficiency, calculated as the percentage of EGFP-positive cells with stable genomic integration (*n* = 4). (**D**) Representative T7 endonuclease I assay gel demonstrating genomic editing efficiency at the *Galns* locus using sgRNA1 and sgRNA2. Indel percentages indicate targeted genomic cleavage efficiency. **** *p* < 0.0001.

To assess gene-editing outcomes in IDLV-transduced fibroblasts, we quantified the percentage of viable cells expressing EGFP by flow cytometry (Figure 2A) [28,29]. UT fibroblasts exhibited background levels of EGFP+ cells (0.01–0.08%). Introduction of IDLV:GALNS (donor-only) resulted in a moderate increase in EGFP+ live cells (mean 3.86 ± 0.63%), reflecting the robust, yet transient, episomal expression that is characteristic of IDLVs [30,31]. Upon the delivery of HDR-competent vectors (IDLV:gRNA1/GALNS and IDLV:gRNA2/GALNS), the proportion of EGFP+ live cells increased significantly (14.12 ± 1.08% and 10.08 ± 0.77%, respectively; **** *p* < 0.0001), indicating efficient gene targeting and cassette knock-in through HDR (Figure 2B). These data showed an additive effect: HDR-driven integration events boosted overall EGFP+ frequency, but episomal donor cassettes provided a meaningful contribution to total EGFP expression, as supported by the persistent signal observed in donor-only conditions.

The calculated HDR efficiency, measured as the percentage of live cells with stable, site-specific integration, was 8.58–12% for IDLV:gRNA1/GALNS and 5.58–7.20% for IDLV:gRNA2/GALNS (Figure 2C). These results were determined by subtracting the percentage of EGFP+ cells in the donor-only condition from the total percentage in the HDR delivery groups, as consistent with the use of EGFP as a sensitive surrogate marker for HDR, and with previously established strategies for quantifying genome-editing outcomes via flow cytometry [28]. The gene editing efficiency of the designed sgRNAs was assessed via the T7 endonuclease I assay. sgRNA1 displayed an editing efficiency of 26.8%, whereas sgRNA2 exhibited 24.6%, confirming their suitability for promoting homologous recombination at the targeted locus (Figure 2D).

These findings demonstrated that the proposed IDLV-mediated gene delivery provided additive gene correction in fibroblasts through transient episomal expression and durable HDR-mediated integration.

### 2.3. IDLV Vectors Drive Robust GALNS Expression and Substrate Reduction in NIH3T3 Cells, with Additive Effects from Donor and HDR Cassettes

**Figure 3:**
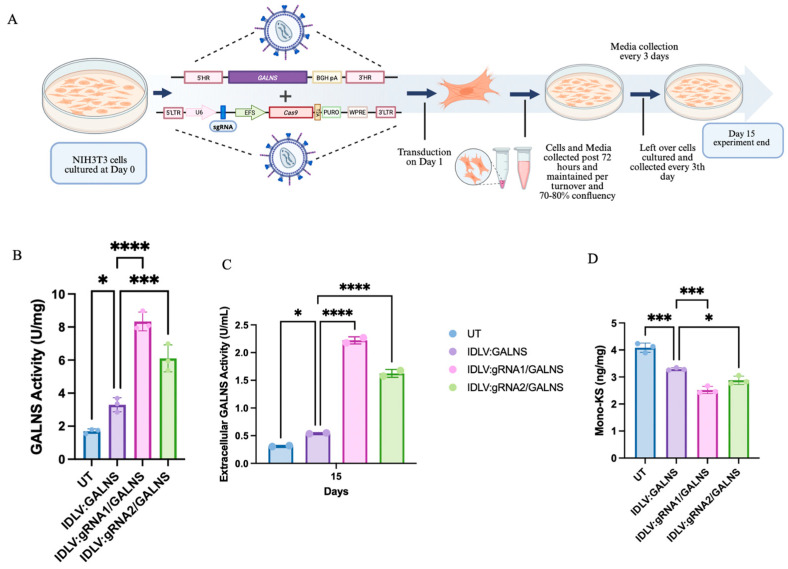
Intracellular and extracellular GALNS activities and KS levels in NIH3T3 cells following 15 days of transduction. (**A**) Schematic experimental approach. NIH3T3 cells were cultured on day 0, transduced on day 1, and collected at multiple time points until day 15 for analyses. This figure was created with Biorender.com. (**B**) Intracellular GALNS enzyme activity measured in NIH3T3 cells after 15 days post-transduction with IDLV:GALNS and HDR-competent vectors (*n* = 3). (**C**) Extracellular GALNS enzyme activity measured in culture media collected from NIH3T3 cells on day 15 post-transduction (*n* = 3). (**D**) Mono-sulfated keratan sulfate (mono-KS) levels measured in NIH3T3 cells post-transduction (*n* = 3). * *p* < 0.05, *** *p* < 0.001, **** *p* < 0.0001.

NIH3T3 cells were transduced with either donor-only IDLV:GALNS or HDR-competent IDLV:gRNA1/GALNS and IDLV:gRNA2/GALNS (Figure 3A). Both intracellular and extracellular GALNS activities, as well as mono-sulfated KS levels, were quantified on day 15 post-transduction. UT NIH3T3 cells exhibited minimal endogenous GALNS activity (1.85 ± 0.19 U/mg, *n* = 3), while the donor only IDLV:GALNS led to a moderate increase (7.83 ± 0.52 U/mg), indicative of substantial episomal expression from the endogenous promoter of the vector. HDR-competent IDLVs conferred a further elevation in the GALNS activity: IDLV:gRNA1/GALNS and IDLV:gRNA2/GALNS produced 11.09 ± 0.78 U/mg and 11.40 ± 0.71 U/mg, respectively (*p* < 0.0001 vs. UT; *p* < 0.01 vs. IDLV:GALNS), reflecting an additive effect of gene-edited and episomal expressions (Figure 3B). Extracellular GALNS activity in culture supernatant was undetectable in UT NIH3T3 cells, low in the IDLV:GALNS group (0.33 ± 0.01 U/mL) and increased significantly after HDR-competent vector treatment (Figure 3C) (IDLV:gRNA1/GALNS: 2.22 ± 0.11 U/mL; IDLV:gRNA2/GALNS: 2.04 ± 0.07 U/mL; *p* < 0.01 vs. IDLV:GALNS, *p* < 0.0001 vs. UT).

Mono-sulfated KS was elevated in UT NIH3T3 cells (4.60 ± 0.63 ng/mg, *n* = 3), reduced in the donor-only group (3.63 ± 0.42 ng/mg), and further reduced in both HDR-competent groups (2.51 ± 0.31 ng/mg for gRNA1; 2.74 ± 0.28 ng/mg for gRNA2; *p* < 0.01 vs. both UT and donor-only, Figure 3D).

These results demonstrated that donor-only IDLV episomes provided robust GALNS expression via an endogenous promoter, while HDR-competent vectors yielded more expression via targeted genomic integration, resulting in additive effects on the enzyme activity and substrate reduction in NIH3T3 cells.

### 2.4. IDLV-Mediated Gene Editing Restores GALNS Activity in MPS IVA Mouse Fibroblasts

**Figure 4:**
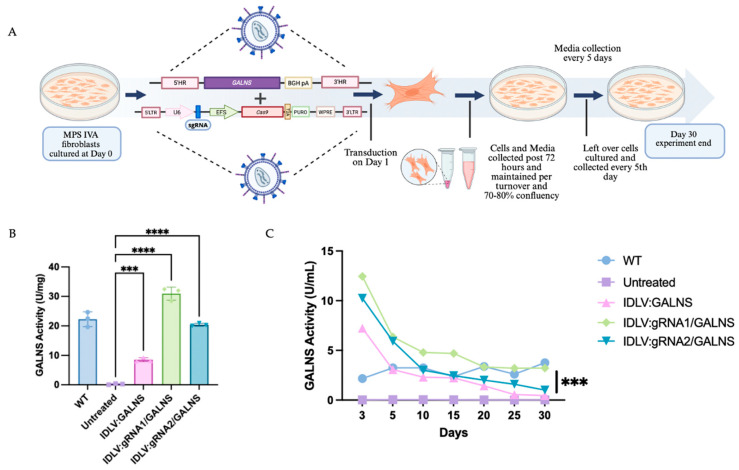
Correction of MPS IVA mouse fibroblasts via IDLV-mediated gene editing. (**A**) Schematic experimental strategy employed to assess the correction of MPS IVA fibroblasts. Cells were cultured on day 0 and transduced with IDLV vectors on day 1. Cells and media were collected at defined intervals up to 30 days post-transduction. This figure was created with Biorender.com. (**B**) Intracellular GALNS enzyme activity in MPS IVA fibroblasts, showing the efficacy of IDLV:GALNS and HDR-competent vectors in restoring enzyme activity (*n* = 3). (**C**) Extracellular GALNS enzyme activities in the media collected from transduced MPS IVA fibroblasts over 30 days (*n* = 3). *** *p* < 0.001, **** *p* < 0.0001.

MPS IVA fibroblasts were transduced with IDLV:GALNS, IDLV:gRNA1/GALNS, or IDLV:gRNA2/GALNS and followed for 30 days to assess both intracellular and secreted GALNS enzyme activity (Figure 4A). As anticipated, UT MPS IVA fibroblasts showed negligible GALNS activity in cell lysates (0.23 ± 0.19 U/mg) and culture media (0.03 ± 0.02 U/mL), while wild-type (WT) control fibroblasts had the enzyme activities of 20.92 ± 0.55 U/mg in lysates and 3.05 ± 0.62 U/mL in media. Cells transduced with donor only IDLV:GALNS achieved partial correction, with mean lysate activity of 14.20 ± 0.87 U/mg and media activity of 1.90 ± 0.83 U/mL, both significantly higher than UT MPS IVA fibroblast levels but still below WT levels (*p* < 0.01). Transduction with HDR-competent IDLV:gRNA1/GALNS and IDLV:gRNA2/GALNS restored the GALNS activity in cell lysates to 31.61 ± 2.13 U/mg and 26.39 ± 2.26 U/mg, respectively, and media activity to 4.43 ± 2.52 U/mL and 3.53 ± 2.07 U/mL (Figure 4B,C). These values approached or exceeded WT, with both groups significantly surpassing donor-only and UT controls (*p* < 0.0001; Figure 4C). To confirm stable integration, we quantified the vector copy number (VCN) per cell (Supplementary Figure S2). The donor-only group showed a low VCN from episomal vector (0.11 ± 0.01), while the HDR-competent groups showed significantly higher VCN (0.34 ± 0.03 for gRNA1 and 0.20 ± 0.01 for gRNA2), confirming successful genomic integration.

These findings confirmed that only HDR-competent IDLV delivery achieved levels of GALNS activity above normal intracellular and extracellular levels in MPS IVA fibroblasts.

### 2.5. IDLV-Mediated GALNS Delivery Rescues Lysosomal Phenotype in Long-Term-Treated MPS IVA Mouse-Derived Fibroblasts

**Figure 5:**
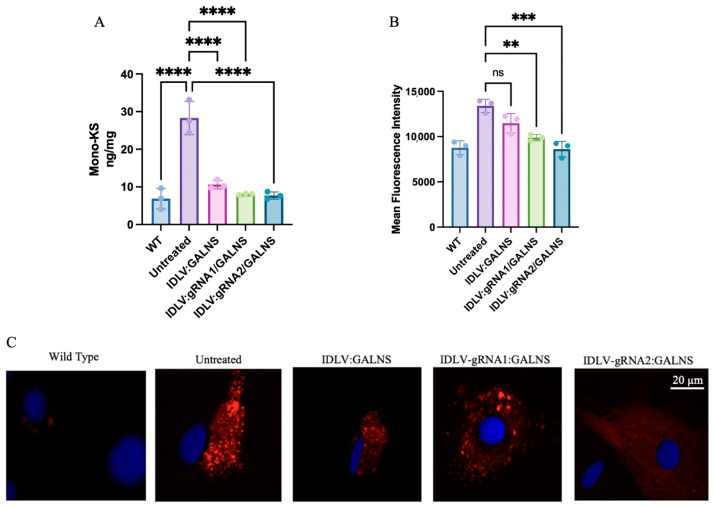
Evaluation of phenotypic correction in MPS IVA fibroblasts following IDLV-mediated gene editing. (**A**) Quantifying mono-sulfated keratan sulfate (mono-KS) levels in MPS IVA fibroblasts after transduction demonstrates substrate reduction across treatment groups (*n* = 3). (**B)** Mean fluorescence intensity (MFI) of lysotracker red dye measured by flow cytometry as a surrogate of lysosomal mass in treated and UT MPS IVA fibroblasts (*n* = 3). (**C**) Representative fluorescence microscopy images of MPS IVA fibroblasts stained with lysotracker red (red) and DAPI (blue) across all treatment groups. Scale bar = 20 μm. ns: nonsignificant, ** *p* < 0.01, *** *p* < 0.001, **** *p* < 0.0001.

To assess long-term phenotypic correction, MPS IVA mouse fibroblasts were transduced with IDLV:GALNS, IDLV:gRNA1/GALNS, or IDLV:gRNA2/GALNS and maintained for 4 weeks in culture. WT and UT MPS IVA mouse fibroblasts served as controls. First, we quantified mono-sulfated KS levels, which are shown in Figure 5A. Intracellular KS levels (*n = 3*) were as follows: WT, 6.87 ± 1.83 ng/mg; UT, 28.33 ± 1.67 ng/mg.mg, IDLV:GALNS 17.60 ± 0.26 ng/mg, IDLV:gRNA1/GALNS 8.03 ± 0.10 ng/mg, and IDLV:gRNA2/GALNS 7.73 ± 0.58 ng/mg. ANOVA was significant (F_4,10_ = 112.7, *p* < 10^−7^). Intracellular KS levels were significantly elevated in UT fibroblasts compared to WT fibroblasts (*p* < 0.0001) and all treated fibroblasts (IDLV:GALNS, *p* < 0.0001; IDLV:gRNA1/GALNS, *p* < 0.0001; IDLV:gRNA2/GALNS, *p* = 0.0005). Intracellular KS levels in the HDR vector-transduced fibroblasts (IDLV:gRNA1/GALNS and IDLV:gRNA2/GALNS) were comparable to WT fibroblasts (ns vs. WT), whereas levels in donor-only transduced fibroblasts remained significantly higher than WT (*p* = 0.01 vs. WT).

Consistent with the substrate reduction, we observed a significant correction of lysosomal pathology. Lysosomal mass (Figure 5B) in UT MPS IVA mouse fibroblasts was measured by mean fluorescence intensity (arbitrary units; *n* = 3 replicates) that yielded: WT 9120 ± 420, UT 14,260 ± 660, IDLV:GALNS 12,400 ± 530, IDLV:gRNA1/GALNS 10,080 ± 610, and IDLV:gRNA2/GALNS 9050 ± 480. One-way ANOVA revealed a significant effect of treatment on fluorescence intensity (F_4,10_ = 18.6, *p* = 0.0003). A post hoc Tukey’s Honest Significant Difference test (Tukey test) showed a significant increase in fluorescence intensity in UT fibroblasts compared to WT fibroblasts (*p* < 0.0001), IDLV:GALNS-transduced fibroblasts (*p* = 0.0007), IDLV:gRNA1/GALNS-transduced fibroblasts (*p* = 0.0042), and IDLV:gRNA2/GALNS-transduced fibroblasts (*p* = 0.03). However, fluorescence intensity did not differ significantly from that of the WT group in any of the treated groups.

Representative fluorescence microscopy images visually confirmed these quantitative findings, showing extensive lysosomal accumulation (intense red staining) in UT cells, partial clearance in the donor-only group, and near-complete resolution of the storage phenotype in the HDR-competent groups (Figure 5C).

These in vitro data demonstrated that IDLV-delivered GALNS, particularly via HDR-competent vectors, achieved sustained enzyme expression, normalized lysosomal storage, and restored the biochemical phenotype of MTOL fibroblasts to WT levels.

B. In vivo validation of the IDLV-mediated CRISPR/Cas9 gene editing strategy

### 2.6. A Single Dose of Dual IDLV Injection Elicits Long-Term GALNS Expression in Plasma and Major Organs

**Figure 6:**
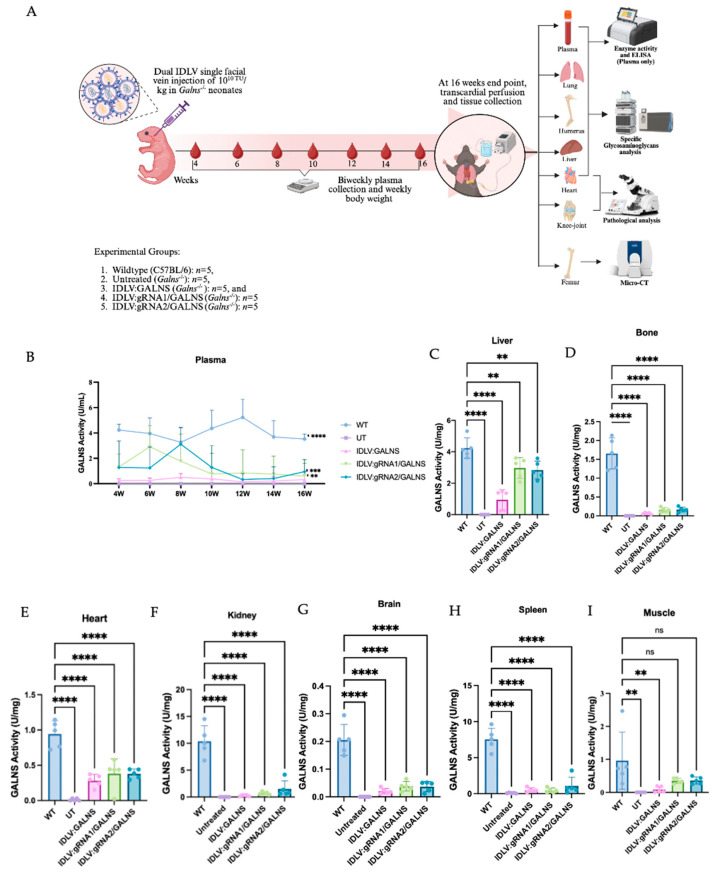
Long-term GALNS enzyme activities in MPS IVA mice following IDLV administration into MPS IVA newborn mice. (**A**) Experimental design of in vivo gene therapy. (**B**) GALNS enzyme activities in plasma samples collected at defined time points over 16 weeks post-treatment. (**C**–**I**) GALNS enzyme activities in liver, bone, heart, kidney, brain, spleen, and muscle at the study endpoint. Each panel represents *n* = 5 mice per group. ns: not significant ** *p* < 0.01, *** *p* < 0.001, **** *p* < 0.0001.

The GALNS activity was monitored for 16 weeks after intravenous delivery of donor only IDLV:GALNS or the HDR-competent vectors IDLV:gRNA1/GALNS and IDLV:gRNA2/GALNS (Figure 6A) at a 10^10^ TU/kg dose into MPS IVA newborn male mice post optimum dose determination. To determine the optimal therapeutic dose for In vivo administration, a dose-ranging study was first conducted in newborn MPS IVA mice. Doses of 1 × 10^10^, 1 × 10^11^, and 1 × 10^12^ TU/kg were administered via facial vein injection (*n* = 3 per group). While the 1 × 10^10^ TU/kg dose was well-tolerated with 100% survival, higher doses resulted in significant toxicity, including failure to thrive, lethargy, and 100% mortality by week 3 for the 1 × 10^12^ TU/kg group and 66% mortality for the 1 × 1011 TU/kg group (Supplementary Figure S3). Therefore, a dose of 1 × 10^10^ TU/kg was selected as the maximum tolerated dose (MTD) for all subsequent efficacy studies. All treated groups showed an increase in body weight over 16 weeks, but this increase did not correlate with the treatment outcome (Supplemental Figure S4). Plasma enzymes first appeared in week 4 and reached a plateau between weeks 8 and 12. By week 16, IDLV:GALNS maintained 0.17 ± 0.13 U /mL, whereas gRNA1 and gRNA2 stabilized at 0.24 ± 0.07 and 0.43 ± 0.05 U/mL, respectively (Figure 6B). Although all treated groups remained <10% of the WT level (5.86 ± 0.62 U/mL), activities were significantly higher than in UT MPS IVA mice, which were consistently undetectable. In tissues, the most significant restoration was observed in the liver (Figure 6C). IDLV:gRNA1/GALNS reached 2.97 ± 0.59 U/mg—74% of WT. IDLV:gRNA2/GALNS achieved a comparable 2.83 ± 0.55 U/mg (67% of WT). The donor-only construct partially increased (0.95 ± 0.53 U/mg, 22% WT). In femur, the GALNS activity increased to 0.38 ± 0.07 U/mg (40% of WT) with both HDR vectors and 0.28 ± 0.08 U/mg^−^ (30% of WT) with IDLV:GALNS (Figure 6D). All vectors delivered detectable enzymes to the myocardium (Figure 6E). HDR constructs produced 0.17 ± 0.06 U/mg (10% of WT). The donor-only group reached 0.06 ± 0.02 U/mg (4% of WT). Although far below physiological levels, these activities matched the partial biochemical and histological corrections documented in cardiac tissue. In kidney, spleen, muscle, and brain, low enzyme activities were detected in kidney (≤13% of WT for gRNA2), spleen (≤22% of WT for gRNA2), skeletal muscle (≤39% of WT for gRNA2) and brain (≤19% of WT for gRNA1), whereas all UT MPS IVA mice remained completely deficient (Figure 6F–I).

Overall, the inclusion of the gRNA cassette enhanced tissue enzyme delivery, elevating hepatic GALNS activity to 70% of WT and providing meaningful activity in the bone, heart, and muscle, while the donor-only vector conferred lower enzyme activity levels.

### 2.7. Mono-Sulfated KS Storage Is Reduced in Plasma, Liver, and Bone After Systemic IDLV Gene Transfer

**Figure 7:**
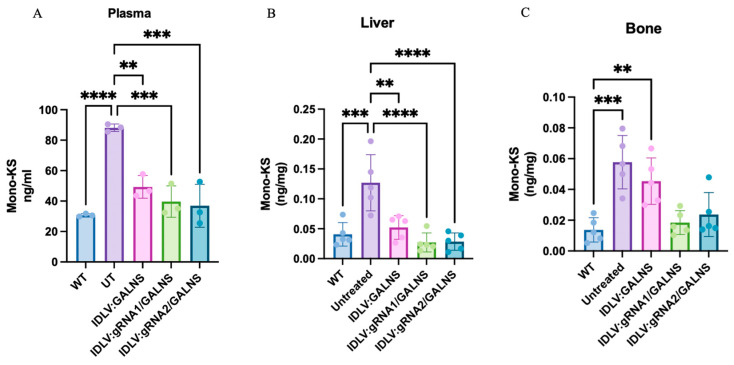
Keratan sulfate reduction in plasma and tissues of MPS IVA mice following IDLV-mediated gene therapy. (**A**) Mono-sulfated keratan sulfate (mono-KS) levels in plasma at the study endpoint from wild-type (WT), untreated (UT), and treated MPS IVA mice, demonstrating systemic substrate reduction. (**B**) Mono-sulfated KS levels in liver at the endpoint. (**C**) Mono-KS levels in the humerus. Each panel represents *n* = 5 mice per group. ** *p* < 0.01, *** *p* < 0.001, **** *p* < 0.0001.

LC-MS/MS was used to quantify mono-sulfated KS in plasma, liver, and humeral bone of WT, UT MPS IVA, and IDLV-treated MPS IVA mice 16 weeks after a single systemic dose of 10^10^ TU/kg (Figure 7A). Data were normally distributed. In plasma KS, UT MPS IVA mice showed a 2.6-fold elevation over WT (145 ± 25 vs. 56.7 ± 3.5 ng/mL, *p* < 0.0001). The donor-only vector lowered KS by 57% (62.8 ± 8.6 ng/mL, *p* < 0.01 vs. UT) but remained higher than WT (*p* < 0.05). IDLV:gRNA1/GALNS (32.4 ± 7.8 ng/mL) and IDLV:gRNA2/GALNS (35.2 ± 7.5 ng/mL) produced a further, significant reduction compared with UT (*p* < 0.0001 and *p* < 0.001, respectively) and were statistically indistinguishable from WT. In liver KS (Figure 7B), UT MPS IVA mice contained three-fold more KS than WT (0.127 ± 0.047 vs. 0.041 ± 0.020 ng/mg). The donor-only vector cut this excess by ~60% (*p* < 0.01 vs. UT), whereas gRNA1 and gRNA2 normalized KS to WT levels (0.027 ± 0.016 and 0.029 ± 0.015 ng/mg; *p* < 0.001 vs. UT). In femur KS (Figure 7C), UT MPS IVA mice harbored a four-fold KS excess over WT (0.058 ± 0.017 vs. 0.014 ± 0.008 ng/mg). IDLV:gRNA1 and IDLV:gRNA2 normalized KS levels to WT values (0.019 ± 0.008 and 0.024 ± 0.014 ng/mg), whereas the donor-only vector delivered a partial, non-significant improvement (0.045 ± 0.015 ng/mg; *p* < 0.01 vs. WT).

UT MPS IVA mice accumulated substantial amounts of mono-sulfated KS in plasma, liver, and bone. A single dose of IDLVs significantly lowered this substrate, with HDR-competent vectors (IDLV:gRNA1/GALNS and IDLV:gRNA2/GALNS) normalizing plasma and liver KS and almost correcting bone KS, while the donor-only vector provided an intermediate biochemical correction.

### 2.8. Systemic IDLV Delivery Mitigates Cardiac Storage but Yields Only Focal Skeletal Benefit

**Figure 8:**
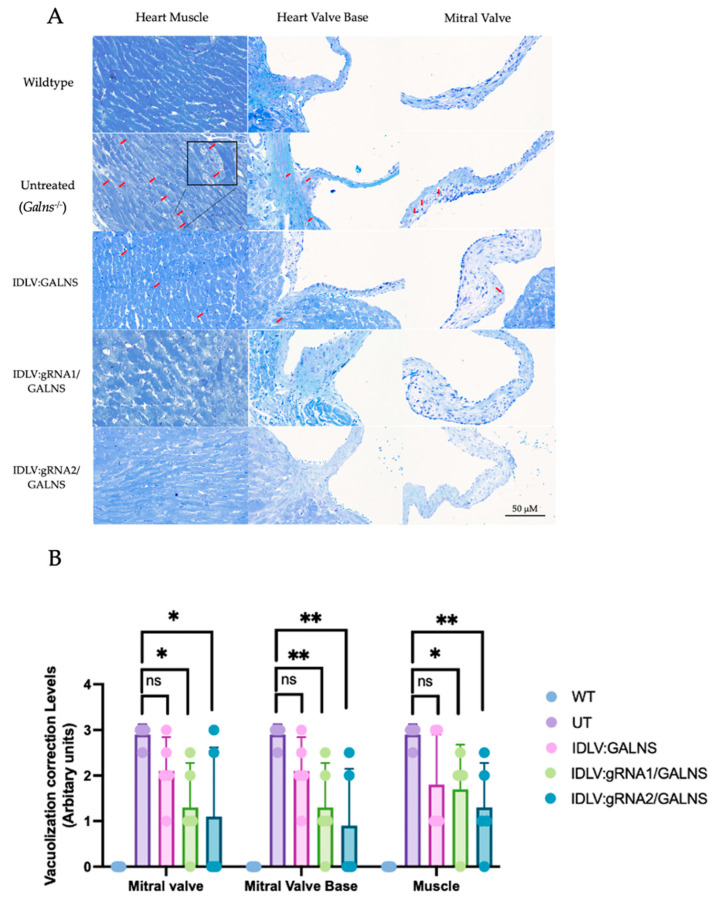

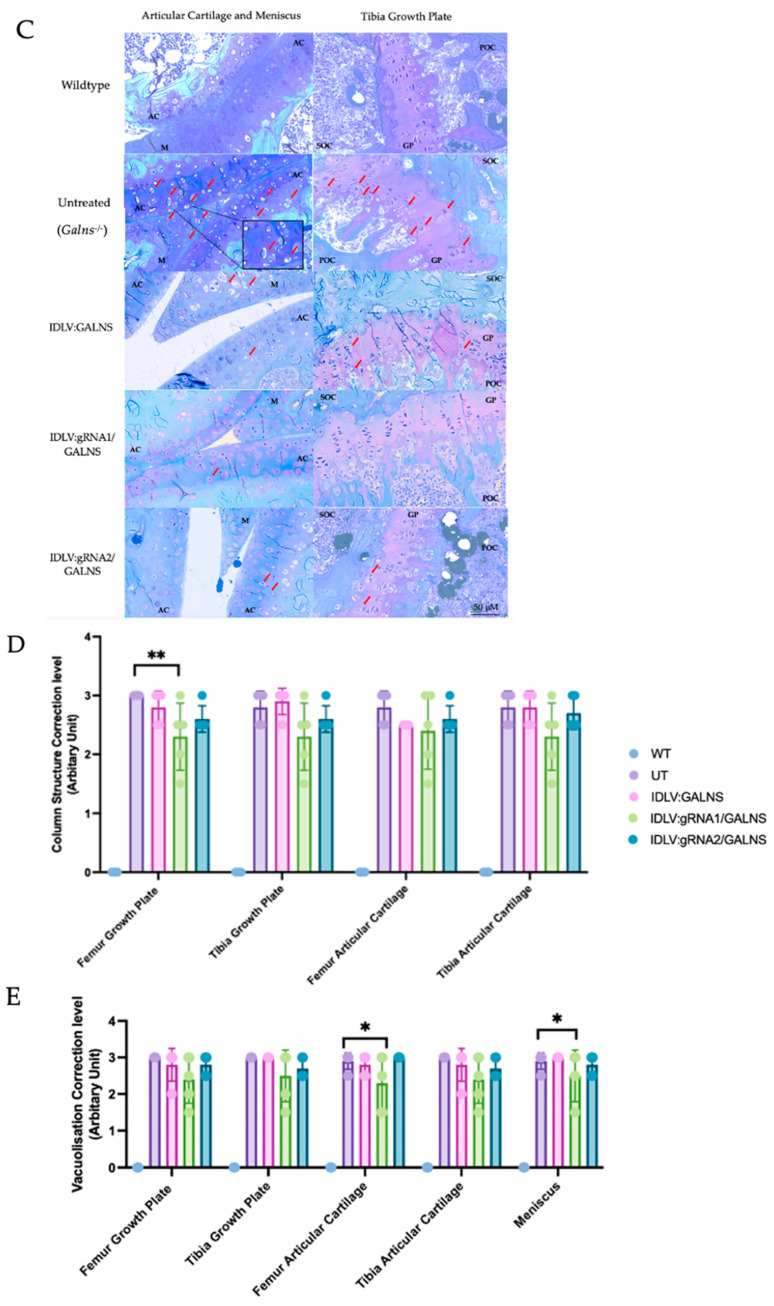
Histopathological correction in cardiac and skeletal tissues following IDLV-mediated gene therapy in MPS IVA mice. (**A**) Toluidine blue staining of heart sections showing representative images of heart muscle, heart valve base, and mitral valve from wild-type, untreated (*Galns*^-/-^), and IDLV-treated groups (IDLV:GALNS, IDLV:gRNA1/GALNS, and IDLV:gRNA2/GALNS). Sections were imaged at 40× magnification; scale bar = 50 μm. Red arrows highlight regions of vacuolization. (**B**) Quantification of vacuolization correction levels in mitral valve, valve base, and heart muscle based on scoring from blinded histological assessment. (**C**) Toluidine blue staining of knee joints showing articular cartilage, meniscus, and tibial growth plate, including the primary ossification center (POC), secondary ossification center (SOC), growth plate (GP), articular cartilage (AC), and meniscus (M). Sections were imaged at 40× magnification; scale bar = 100 μm. Red arrows indicate chondrocyte vacuolization and disorganized structure. (**D**) Quantification of growth plate column structure correction in the femur and tibia. (**E**) Quantification of chondrocyte vacuolization levels in the femur, tibia, and meniscus. All data are expressed as mean ± SD. Statistical significance was determined by one-way ANOVA with Tukey’s post hoc test. ns: not significant, * *p *< 0.05, ** *p *< 0.01.

Toluidine-blue staining of heart coronal sections revealed severe vacuolization in all cardiac compartments of UT MPS IVA mice (UT; 2.90 ± 0.05 Arbitrary Unit (AU)), whereas WT hearts were lesion-free (0.00 ± 0.00 AU; Figure 8B). Mice that received IDLV carrying the *GALNS* donor alone (IDLV:GALNS) or donor + HDR cassettes (IDLV:gRNA1/GALNS or IDLV:gRNA2/GALNS) showed lower mean scores (1.73 ± 0.24, 1.95 ± 0.31, and 1.18 ± 0.32 AU, respectively) (Supplementary Table S1), yet none of the treated cohorts differed significantly from UT in Dunn’s post-hoc testing after a global Kruskal-Wallis difference was confirmed (H = 46.7, *p* < 0.0001). Thus, at this single dose and 16 weeks old, IDLV administration trended toward correction but did not achieve statistical normalization of cardiac lesions.

Composite scores across five regions (growth plates, articular cartilage, meniscus, ligament) of sagittal knee-joint sections echoed the cardiac pattern, with UT mice displaying a heavy storage burden (2.96 ± 0.08 AU) and WT mice remaining normal (0.00 ± 0.00 AU; Figure 8D). Among treated groups, only IDLV: gRNA1/GALNS produced a significant reduction (2.42 ± 0.25 AU; –18% vs. UT, Dunn’s *p* = 0.049), whereas donor-only and gRNA2 vectors afforded smaller, non-significant changes (2.88 ± 0.16 and 2.80 ± 0.14 AU, respectively). The global Kruskal-Wallis comparison was again significant (H = 82.8, *p* < 0.0001), driven primarily by the WT-versus-disease contrast. Disorganization of chondrocyte columns in UT knees averaged 2.85 ± 0.10 AU (Figure 8E). HDR-competent vectors lowered the mean to 2.33 ± 0.26 AU (gRNA1; –18%) and 2.63 ± 0.12 AU (gRNA2; –8%), but neither difference reached significance after correction (Dunn’s *p* > 0.05). Donor-only treatment was indistinguishable from UT (2.75 ± 0.18 AU) (Supplementary Table S2).

It is noteworthy that we observed significant inter-animal variability in the degree of histological correction, particularly in cardiac and skeletal tissues (Figure 8B,D,E). This finding highlights a common challenge in preclinical gene therapy studies and underscores the influence of complex biological factors, which will be further described in the Discussion.

A single systemic administration of IDLV at 10^10^ TU/kg attenuated cardiac vacuolization and provided a statistically significant, though partial, reduction in storage lesions in knee cartilage when the gRNA1 HDR construct was used. Improvements in heart tissue and growth-plate architecture did not achieve statistical significance, indicating that higher vector doses, extended follow-up, or combinatorial approaches may be required for complete histological correction in the MPS IVA mouse model.

### 2.9. Bone Morphometric Analysis Reveals Selective, Modest Improvements After Systemic IDLV Administration

**Figure 9:**
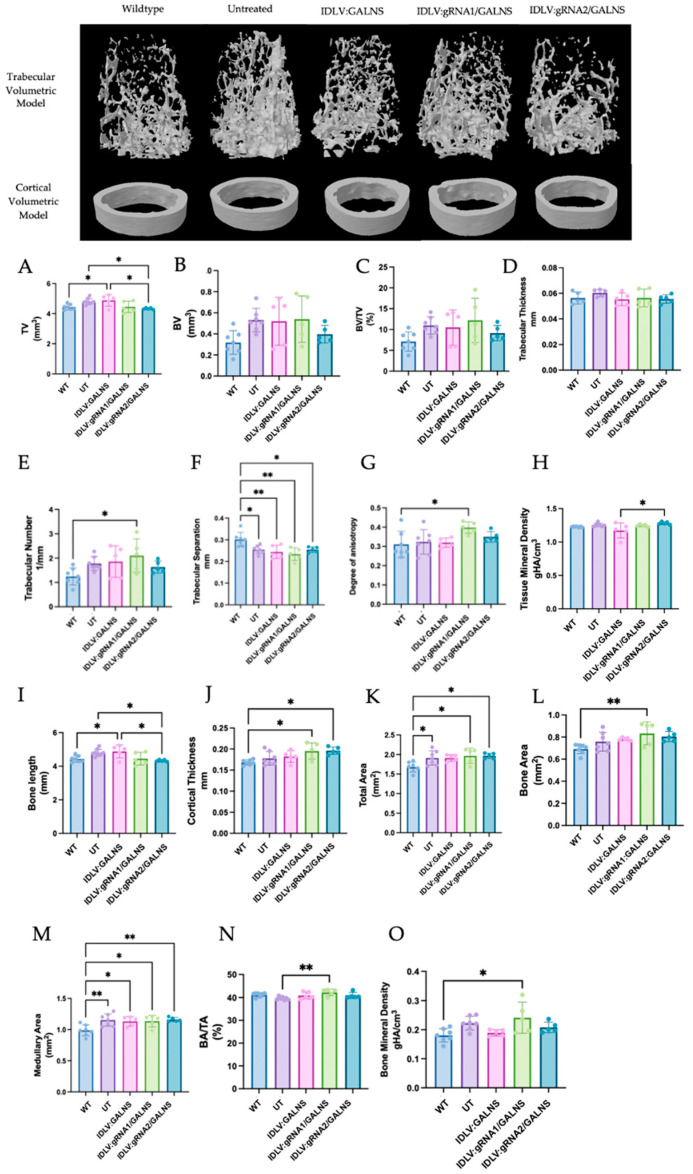
Bone morphometric evaluation in MPS IVA mice following IDLV gene correction. Representative 3D volumetric reconstructions of trabecular (**top**) and cortical (**bottom**) bone structures are shown for each group. (**A**–**J**) Trabecular parameters including total volume (TV), bone volume (BV), bone volume fraction (BV/TV), trabecular thickness (Tb.Th), trabecular number (Tb.N), trabecular separation (Tb.Sp), degree of anisotropy (DA), and tissue mineral density (TMD). (**I**–**O**) Cortical bone metrics include bone length, cortical thickness (Ct.Th), total area (TA), bone area (BA), medullary area (MA), bone area fraction (BA/TA), and cortical bone mineral density (Ct.BMD). One-way ANOVA with Tukey test. Comparisons were made between UT and WT groups; * *p* < 0.05, ** *p* < 0.01.

Representative 3D micro-CT reconstructions of the distal femoral metaphysis are shown in Figure 9 (Top). UT MPS IVA mice exhibited the rarefied trabecular lattice and thin, irregular cortical shell. All IDLV-treated groups appeared qualitatively denser than UT, but none fully recapitulated the compact WT architecture.

Quantitative trabecular metrics (Figure 9A–H) were analyzed. One-way ANOVA identified significant heterogeneity for total trabecular volume (TV; *p* = 0.0026), trabecular separation (Tb.Sp; *p* = 0.0013), and tissue mineral density (TMD; *p* = 0.020). TV was 8% higher in UT than WT (4.80 ± 0.20 mm^3^ vs. 4.44 ± 0.19 mm^3^, *p* < 0.05). IDLV:gRNA1/GALNS normalized TV to WT (4.45 ± 0.38 mm^3^; *p* > 0.05 vs. WT; *p* = 0.13 vs. UT). IDLV:gRNA2/GALNS produced a statistically significant 10% reduction relative to UT (4.33 ± 0.05 mm^3^; Tukey test, *p* = 0.022). The donor-only vector yielded a moderate, non-significant decrease. Tb.Sp was significantly lower in UT (0.255 ± 0.019 mm) than in WT (0.301 ± 0.033 mm; Tukey test, *p* = 0.021). All three vectors trended upward (0.244–0.255 mm), but none restored the WT value (*p* > 0.05 vs. UT). TMD decreased slightly with the donor-only vector (1.17 ± 0.11 g/HA cm^3^) and increased modestly with IDLV:gRNA2/GALNS (1.28 ± 0.02 g/HA cm^3^). Although the overall ANOVA was significant, no single pair-wise contrast survived multiplicity correction.

Cortical parameters (Figure 9I–O), Significant group effects were observed for bone length (BL; *p* = 0.046), cortical thickness (Ct.Th; *p* = 0.0071), total area (TA; *p* = 0.0080), bone area (BA; *p* = 0.0137), medullary area (MA; *p* = 0.0053) and BA/TA ratio (*p* = 0.014). Bone length was greater in UT than in WT (4.65 ± 0.16 mm vs. 4.30 ± 0.10 mm, *p* < 0.05). All three IDLV treatments shortened the femur significantly relative to UT (IDLV:GALNS 4.49 ± 0.07 mm; IDLV:gRNA1/GALNS 4.46 ± 0.14 mm; IDLV:gRNA2/GALNS 4.38 ± 0.08 mm; all Tukey test, *p* < 0.05 vs. UT), yet lengths remained above the WT mean. Ct.Th peaked in UT (0.242 ± 0.009 mm vs. 0.225 ± 0.009 mm in WT, *p* < 0.05). IDLV:GALNS (0.205 ± 0.017 mm) and IDLV:gRNA2/GALNS (0.200 ± 0.014 mm) showed a significant reduction vs. UT (Tukey test, *p* < 0.05) but did not fully normalize; IDLV:gRNA1/GALNS was intermediate (0.231 ± 0.015 mm; *p* = 0.06 vs. UT). TA, BA, and MA were all enlarged in UT. Each index declined slightly in the treated cohorts, reaching significance for TA and BA with gRNA1 and gRNA2 vectors (*p* < 0.01) and for MA with every vector (*p* ≤ 0.05). However, the BA/TA ratio remained elevated, indicating persistent cortical thinning relative to bone size.

Micro-CT analysis at 16 weeks showed that IDLV:gRNA1/GALNS corrected total trabecular volume and partially rescued cortical overgrowth, while IDLV:gRNA2/GALNS additionally improved mineral density and cortical thickness. Despite these gains, most trabecular quality indices (BV/TV, Tb.Th, Tb.N, and DA) and cortical measures (BA/TA and TMD) remained abnormal. Thus, a single systemic dose of 10^10^ TU/kg delivered modest, site-specific skeletal benefits, emphasizing the need for higher dosing, longer follow-up, or combinatorial approaches to comprehensively reverse the bone phenotype in MPS IVA mice.

### 2.10. IDLV-Based CRISPR Delivery Does Not Elicit Chronic Hepatic or Systemic Toxicity in MPS IVA Mice

**Figure 10:**
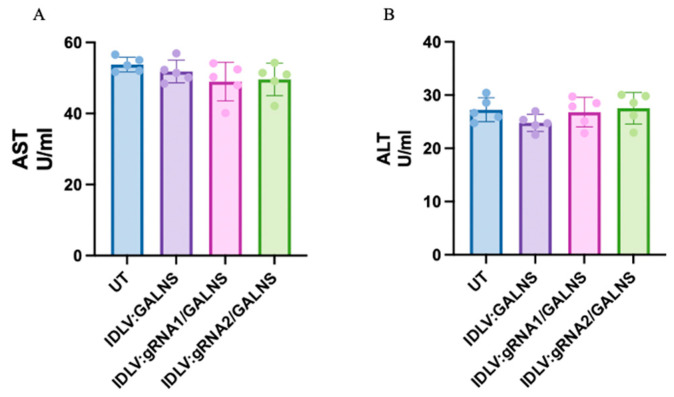
Evaluation of liver toxicity biomarkers in IDLV-treated mice. (**A**) Aspartate aminotransferase (AST) and (**B**) alanine aminotransferase (ALT) levels were quantified in plasma from each group at the experimental endpoint (*n* = 5). No significant elevation in liver enzymes was observed.

Before evaluating long-term therapeutic efficacy, we examined whether the systemic administration of our IDLVs provoked hepatic or systemic injury. Sixteen-week-old MPS IVA mice received either (i) IDLV:GALNS (donor only), (ii) IDLV:gRNA1/GALNS, or (iii) IDLV:gRNA2/GALNS at 10^10^ TU/kg dose; UT littermates served as controls (n = 5 per group). Sixteen weeks post-injection, serum aspartate aminotransferase (AST) (systemic) and alanine aminotransferase (ALT) (liver-specific) activities were quantified. As depicted in Figure 10A, mean AST values remained within the physiological range (50–55 U/mL) and were indistinguishable between UT (54.2 ± 1.6 U/mL) and treated groups (IDLV: GALNS 52.8 ± 2.3; IDLV: gRNA/ALNS 50.4 ± 3.8; IDLV:gRNA2/GALNS 52.1 ± 2.5 U/mL; one-way ANOVA, *p* > 0.05). A similar pattern was observed for ALT (UT 27.1 ± 1.3 U/mL vs. 25.7 ± 1.9, 25.9 ± 2.1 and 26.8 ± 1.7 U/mL, respectively; *p* > 0.05) (Figure 10B). Collectively, these findings demonstrated that the dosing regimen of 10^10^ TU/kg IDLV carrying either donor or HDR components was well-tolerated and failed to induce chronic hepatic or systemic toxicity in adult MPS IVA mice.

### 2.11. Humoral Immunity Against Cas9 and GALNS Remains Low After IDLV Treatment

To determine whether IDLV-delivered editing machinery provokes an unwanted humoral response, we quantified circulating antibodies directed against human Cas9 (hCas9) and the therapeutic GALNS enzyme 16 weeks after vector administration. Sera from UT MPS IVA, WT, and treated mice with IDLV:GALNS (donor only), IDLV: gRNA1/GALNS, or IDLV:gRNA2/GALNS (*n* = 5 per group) were analyzed by ELISA.

**Figure 11:**
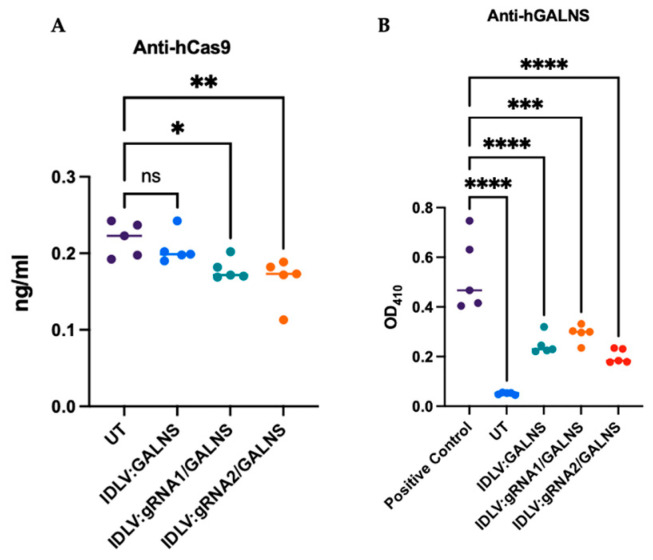
Antibody-mediated immune response in IDLV-treated mice. (**A**) Anti-Cas9 antibody levels measured in plasma samples, indicating host immune response against the Cas9 protein (*n* = 5). (**B**) Anti-GALNS antibody levels by ELISA, using positive control sera from enzyme replacement-treated MPS IVA mice (*n* = 5). * *p* < 0.05, ** *p* < 0.01, *** *p* < 0.001, **** *p* < 0.0001.

Anti-hCas9 IgG (Figure 11A). baseline anti-hCas9 titers in UT MPS IVA mice averaged ~0.22 ng/mL and did not rise in any treated cohort. The pre-existing immunity to WT Cas9 in mice has been demonstrated, consistent with the results [23,32,33]. Indeed, mice receiving the complete HDR vectors (IDLV:gRNA1 or IDLV:gRNA2) showed slightly lower mean values (0.15–0.17 ng/mL), whereas IDLV:GALNS remained indistinguishable from the controls. These results indicated that, even though hCas9 was transiently expressed from episomal forms, the dose used here failed to elicit a systemic anti-Cas9 response.

Anti-human GALNS IgG (Figure 11B). Antibody binding to recombinant human GALNS was minimal in the IDLV:GALNS group (OD_410_ = 0.05). Vectors containing both donor and gRNA cassette produced moderate titers (OD_410_ = 0.25–0.30) that remained significantly below those of UT MPS IVA mice (OD_410_ = 0.42; *p* < 0.001) and significantly lower than a positive-control cohort actively immunized with human GALNS protein (OD_410_ = 0.80; *p *< 0.0001). Thus, the long-term expression of the transgene or the presence of genome-editing components does not trigger a robust anti-human GALNS response.

Collectively, these data demonstrated that systemic delivery of 10^10^ TU/kg IDLV carrying either donor alone or HDR reagents was well-tolerated at the humoral level, with no appreciable increase in anti-Cas9 antibodies and only low-to-moderate, sub-pathological anti-human GALNS antibody titers.

## 3. Discussion

The development of a safe and durably effective therapy for MPS IVA is a pressing need, considering the severe systemic manifestations and the limitations of current treatments such as ERT and HSCT [34,35]. While gene therapy, particularly approaches utilizing CRISPR/Cas9 for precise genome editing, holds promise, this study takes a step further. It explores an innovative strategy that could potentially revolutionize the field of gene therapy. By harnessing the potential of IDLVs, we have uncovered a robust delivery platform that could deliver a functional GALNS enzyme, aiming to achieve therapeutic benefit through both transient episomal expression and stable, HDR-mediated genomic integration upstream of the endogenous murine *Galns* promoter. Our findings underscore the potential of IDLVs not just as a delivery platform but as a game-changer in the field of gene therapy, demonstrating significant biochemical correction in vitro and promising, albeit partial, therapeutic effects in vivo.

The IDLV system, as highlighted in this work, has the potential to mediate substantial *GALNS* expression through two distinct mechanisms. The donor-only IDLV: GALNS vector led to significant GALNS enzyme activity, confirming robust expression from an episomal vector form, consistent with prior studies demonstrating the utility of IDLVs for transient, yet therapeutically relevant, protein production [30,31]. The incorporation of CRISPR/Cas9 components to facilitate HDR (IDLV:gRNA1/GALNS and IDLV:gRNA2/GALNS) resulted in an additive effect, significantly boosting both intracellular and secreted GALNS levels beyond those achieved by episomal expression alone. In vitro, this dual action normalized the GALNS activity, resulting in a substantial clearance of mono-sulfated KS in MPS IVA fibroblasts, with calculated HDR efficiencies ranging from 12.6% to 15.7%. These efficiencies proved sufficient for profound biochemical rescue, underscoring the potency of combining even moderate stable integration with the background of episomal expression. The IDLV system offers a favorable safety profile by minimizing the risk of insertional mutagenesis associated with integrating LVs [14,26,27].

This foundational study successfully established the In vivo therapeutic potential and transduction capabilities of the IDLV platform. The logical next step, which will be the focus of a forthcoming manuscript, is the detailed in vitro molecular characterization and safety profiling of the editing machinery. This future work will include the comprehensive on- and off-target analyses that are essential for clinical translation. A key conceptual feature of our approach is the uncoupling of vector delivery from genomic integration. By using an IDLV, we mitigate the risks associated with random integration that conventional LVs present. Instead, integration is directed with high precision by the CRISPR/Cas9 system, which induces a site-specific DSB and co-opts the cell’s HDR machinery to use the delivered IDLV genome as a donor template. Our VCN data, showing significantly higher copy numbers only in the presence of Cas9 and sgRNA, strongly support this proposed mechanism and highlight a significant safety advantage of this platform. Our primary goal was to demonstrate the feasibility of the dual-IDLV system and, most importantly, its ability to achieve functional therapeutic correction. It is critical to carefully evaluate the efficiency of HDR. To this end, we employed the EGFP reporter system, a widely used and accepted strategy for rapidly assessing successful knock-in events that result in stable transgene expression. The logic of subtracting the donor-only EGFP signal, while an approximation, provides a reasonable estimate of the additional, stable expression derived from CRISPR/Cas9-mediated integration, distinguishing it from the baseline episomal expression. The limitation of the current study is that comparing the GFP-positive ratio between the donor-only and donor + Cas9 groups is not a precise method for assessing the HDR efficiency. We will perform additional well-established assays, such as junction PCR, to evaluate HDR, including its efficiency and the types of editing outcomes. This potential of the IDLV system should instill optimism about the future of gene therapy for MPS IVA.

Translating these in vitro benefits to an In vivo setting yielded encouraging results. A single systemic administration of IDLVs to MPS IVA newborn mice led to long-term GALNS expression in plasma and, importantly, in several target organs, including the liver (up to 74% of WT levels with HDR vectors), bone (up to 40% of WT), and heart (up to 10% of WT). Persistent but sub-physiological plasma activity suggests that most enzymes remain intracellular, consistent with robust corrections in highly vascularized organs and partial improvements in tissues with limited vascular access. This discrepancy is likely due to the highly permeable, fenestrated endothelium of the liver, which allows for superior vector uptake, whereas the continuous capillaries of the heart muscle form a significant barrier to systemic delivery [36,37]. This widespread enzyme distribution was translated into significant reductions in KS storage in plasma, liver, and bone with HDR-competent vectors, demonstrating favorable therapeutic efficacy by normalizing KS in plasma and liver and nearly correcting it in bone. These In vivo data compared favorably with the challenges often faced by other viral vectors, such as AAV-based liver-targeted therapies that also show partial skeletal and cardiovascular correction [3,38], including payload capacity and pre-existing immunity in AAVs [22,36], and concerns regarding random integration in conventional LVs [25]. Briefly, this IDLV-based strategy overcomes several critical limitations of adeno-associated virus (AAV) vectors. The IDLV’s large packaging capacity (~8–10 kb) is essential for delivering the complex CRISPR/Cas9 machinery used in this study, which is impossible with size-limited AAVs (~4.7 kb) [38]. Furthermore, its VSV-G pseudotype bypasses the widespread pre-existing immunity that restricts the clinical use of many AAV serotypes [39]. Most importantly, this approach provides a superior safety profile by uncoupling vector delivery from genomic integration. Unlike conventional LVs that integrate randomly or AAV episomes that can be lost in dividing cells, our system uses CRISPR/Cas9 to direct the integration of a non-integrating vector. This strategy provides the durable correction of an integrating vector but without the associated risk of random insertional mutagenesis [40]. Our study contributes to a rapidly advancing field where diverse strategies are being pursued to treat MPS IVA. Recent efforts have focused on overcoming the significant challenge of delivering therapies to avascular tissues, demonstrating progress in AAV-mediated gene therapy aimed at enhancing skeletal and cardiac tissue correction [41,42,43]. Further work has also explored the viability of non-viral, nanoparticle-based delivery systems to bypass vector-associated limitations [20]. The observed low immunogenicity against Cas9 and GALNS in our study, a significant finding, further supports the tolerability of this IDLV-based approach, potentially reducing the risk of immune rejection in patients, a common concern for viral vector-based therapies [44].

Despite these successes, the study also identified clear areas for future optimization. While biochemical correction was substantial, plasma GALNS activity remained subphysiological (<10% of WT), and the phenotypic rescue in skeletal and cardiac tissues was partial at the 16-week endpoint with the current 1 × 10^10^ TU/kg dose. For instance, IDLV:gRNA1/GALNS significantly reduced knee cartilage storage lesions and normalized total trabecular volume, and IDLV:gRNA2/GALNS improved tissue mineral density and cortical thickness. However, comprehensive normalization of cardiac lesions was not achieved, and several bone morphometric parameters remained abnormal. This mirrors the challenges encountered in other gene therapy attempts for MPS disorders, where achieving full correction in difficult-to-target tissues, such as cartilage and heart valves, remains a significant hurdle [43,44,45]. This challenge is also noted in AAV8-mediated gene therapies for MPS IVA [46]. These tissues often exhibit unique avascular or hypovascular structural characteristics, rendering them less amenable to gene therapy and other therapeutic approaches. Our partial success in these areas is a significant step forward.

A notable finding in our In vivo studies was the significant inter-animal variability in therapeutic response, particularly in the histological correction of cardiac and skeletal tissues (Figure 8). This observation is consistent with challenges reported in other preclinical gene therapy studies and likely reflects the complex interplay of multiple biological factors. Potential sources for this variability include: (i) subtle differences in vector biodistribution following systemic neonatal injection, leading to varied transduction efficiencies in target tissues like cartilage and heart valves; (ii) individual variations in the host immune response to the vector capsid and/or the human GALNS transgene, which could affect the persistence of transduced cells and the activity of the secreted enzyme; and (iii) the intrinsically stochastic nature of the CRISPR/Cas9 editing process itself, which can lead to different ratios of HDR and indel events among animals. Acknowledging and understanding these sources of variability is critical for the future clinical translation of this approach and underscores the need for strategies that ensure more uniform and robust vector delivery and expression [47,48,49].

To advance this IDLV-CRISPR/Cas9 approach towards clinical relevance, future research must focus on enhancing the IDLV vector, improving HDR efficiency, and validating IDLV-based ex vivo strategies. Firstly, the design of the IDLV template for enhanced episomal expression and stability is critical. This involves several considerations: (1) Optimized Expression Cassettes: We will incorporate stronger, ubiquitously acting promoters (e.g., CAG, EF1α, PGK) or potentially cell-type-specific promoters if refined targeting is desired within the IDLV backbone to drive higher and more sustained *GALNS* gene expression in an episomal form [50,51,52]. (2) Prolonging Episomal Persistence and Mitigating Silencing: IDLV episomes can be lost during cell division or silenced over time. Strategies to counteract this include incorporating Scaffold/Matrix Attachment Regions (S/MARs) into the IDLV genome to tether episomes to the nuclear matrix, potentially improving episomal maintenance and stabilizing transgene expression [53,54]. Flanking the expression cassette with insulator elements (e.g., cHS4), which are primarily used in integrating vectors [55], might help organize IDLV episomes into independent chromatin domains, potentially reducing silencing and ensuring more consistent expression [56]. The potential utility of retroviral elements with insulator properties, such as IS2, in episomal contexts could also be further explored. (3) Vector Particle Engineering and Evasion of Innate Immunity: Pseudotyping IDLVs with alternative viral envelopes or incorporating targeting ligands can enhance transduction of specific cell types or reduce pre-existing immunity [57,58]. Modifying IDLV components or co-delivering immunomodulatory factors to reduce innate immune sensing of viral RNA/DNA can also improve vector persistence and safety [59].

Secondly, improving IDLV-mediated HDR efficiency is vital, as the IDLV genome serves as the donor template. Key strategies include: (1) Optimized IDLV Donor Template, ensuring the structural integrity, efficient nuclear delivery, and sustained availability of the IDLV donor template during DSB induction is critical. The design elements discussed for enhancing episomal expression also contribute to the quality of the donor template. (2) Co-delivery of HDR-Enhancing Factors via IDLV systems can be engineered to transiently express factors that bias DNA repair towards HDR or inhibit NHEJ. This could involve IDLVs encoding dominant-negative NHEJ proteins, shRNAs against key NHEJ factors (e.g., Lig4, DNA-PKcs) [29,60], or delivering proteins/mRNAs for HDR-promoting factors like Rad51 variants [29,61,62,63]. (3) Advanced Nuclease Systems Delivered by IDLVs: The large packaging capacity of IDLVs makes them suitable for delivering more complex gene editing systems, like base or prime editors, for direct correction of specific *GALNS* mutations [64,65]. (4) Coordinated Delivery and Expression: Optimizing the stoichiometry and timing of nuclease expression and donor template availability is crucial, building on work demonstrating IDLV feasibility for co-delivering ZFNs and donor templates [66].

Thirdly, optimizing the In vivo efficacy of IDLV-based therapies requires attention to several factors, including Dose Escalation and Delivery Routes. Given the favorable safety profile observed, careful dose-escalation studies for systemically administered IDLVs are warranted. Furthermore, considering alternatives to WT Cas9 is crucial for refining the safety and specificity of genome editing, particularly for clinical translation. Of particular relevance is the exploration of nickase Cas9 (nCas9). Unlike WT Cas9, nCas9 creates only single-strand breaks in the DNA. When used in pairs with two guide RNAs targeting nearby sites on opposite strands, nCas9 can generate a double-strand break with significantly reduced off-target activity compared to standard Cas9, enhancing overall safety [20,21,67]. This paired nCas9 approach can facilitate the efficient HDR, which is paramount for precise gene insertion, directly supporting the core mechanism of our IDLV-based *GALNS* gene delivery strategy. Exploring these advanced nuclease systems within the IDLV delivery platform holds substantial promise for overcoming current challenges in gene editing for MPS IVA by improving precision and enhancing the overall safety profile.

Building upon our current efficacy for *GALNS* delivery via IDLV-CRISPR/Cas9, a pivotal next step is its translation to an ex vivo gene therapy strategy using patient-derived hematopoietic stem and progenitor cells (HSPCs) and mesenchymal stem cells (MSCs). This requires rigorous validation, including efficient IDLV-mediated transduction and targeted *GALNS* integration in CD34+ HSPCs and MSCs, ensuring stable, supraphysiological enzyme secretion [68]. Crucially, robust in vitro cross-correction of MPS IVA cellular phenotypes by these IDLV-edited cells must be confirmed [68]. Comprehensive genotoxicity assessments (off-target analysis, clonal tracking) with long-term In vivo studies in humanoid animal models (evaluating systemic enzyme distribution, pathology correction, and engraftment) are imperative for advancing this IDLV-based approach to clinical application [69,70].

## 4. Materials and Methods

### 4.1. Mammalian Cell Culture

Mouse NIH3T3 cells (skeletal dysplasia biobank, Wilmington, DE, USA) were maintained in complete Dulbecco’s modified Eagle’s medium nutrient mixture F-12 (DMEM/F12, Gibco#11320033, Grand Island, NY, USA) supplemented with 10% fetal bovine serum (FBS; Gibco#10082147, Grand Island, NY, USA), 1% streptomycin/penicillin (Penicillin-Streptomycin, Gibco#15070063, Grand Island, NY, USA). MPS IVA knock-out mouse-derived fibroblasts were cultured in complete Dulbecco’s modified Eagle’s medium nutrient mixture F-12 supplemented with 20% fetal bovine serum, 1% streptomycin/penicillin. All the cells were incubated at 37 °C in 5% CO_2_.

### 4.2. Integrase Deficient Viral Vector (IDLV) Construction

#### 4.2.1. Design of IDLV Delivering Cas9 and sgRNA

Two sgRNAs: gRNA1- AGCAAACCAAGCTCCCGCTGAGG and gRNA2- AGCGGGAGCTTGGTTTGCTATGG were designed by Benchling to target the upstream of the ATG initiation codon of exon 1 of the *Galns* locus. IDLV was designed with the U6 promoter driving sgRNA expression and the EFS promoter for the Cas9 expression. Vectors used in the in vitro and in vivo experiments were synthesized by VectorBuilder Inc. (Chicago, IL, USA)

#### 4.2.2. Design of IDLV Delivering Expression Cassette

The IDLV was designed with two homologous arms to the mouse *Galns* locus, approximately 800–900 bp long, flanking the human *GALNS* cDNA and a bovine growth hormone (bGH) poly(A) signal sequence. For additional experiments, IDLV carrying the enhanced green fluorescent protein (*EGFP*) gene downstream of the *GALNS* cDNA, with the T2A sequence for ribosome skipping, was used. Vectors used in the in vitro and In vivo experiments were synthesized by VectorBuilder Inc. (Chicago, IL, USA). All vectors were supplied as In vivo-grade, purified preparations. According to the manufacturer’s quality control, all lots possessed a functional titer of >1 × 10^9^ transducing units (TU)/mL, as determined by transduction of HEK293T cells and subsequent qPCR analysis and were certified to be free of bacterial and mycoplasma contamination.

### 4.3. IDLV Transduction

The transduction efficacy was evaluated on NIH3T3 cells with different MOIs at 10, 15, and 20. Briefly, cells were cultured at a seeding density of 10^5^ cells/mL in 12-well Corning culture plates (Corning, NY, USA). After 24 h of incubation, the cells were transduced with IDLV at different MOIs. After 48 h of transduction, the media were refreshed, and the cells were collected 72 h post-transduction for further analysis. Following optimal MOI determination, the transduction ratio was determined by transducing NIH3T3 cells at a concentration of 10^5^ cells/mL. The IDLV:GALNS and IDLV: Cas9/sgRNA1 were used to transduce NIH3T3 cells at 1:1, 1:2, 1:2.5, and 2:1 ratios, with an experimental flow similar to MOI determination, where cells were collected 72 h post-transduction for further analysis. A 15-day experiment was conducted following the determination of MOI and transduction ratio to assess the persistent expression of the delivered cassette. To evaluate post-transduction efficiency and the rate of Homology-Directed Repair (HDR), cells were transduced with an EGFP-containing donor IDLV. We then estimated HDR efficiency using an EGFP reporter system and flow cytometry. By quantifying the percentage of EGFP-positive cells in HDR-competent groups and subtracting the baseline EGFP expression from the donor-only (episomal) condition, we could estimate the contribution from HDR-mediated events. For a proof-of-concept study, this high-throughput approach provides a functionally relevant assessment of overall correction. However, we acknowledge that this is an indirect measure, and future studies will utilize direct molecular assays, such as junction PCR or targeted deep sequencing, for precise quantification.

For long-term evaluation, MPS IVA knock-out mouse-derived fibroblasts were similarly treated and monitored over 30 days. During this period, cells were collected every five days, and the media were changed every three days post-transduction for further analysis.

### 4.4. On-Target Evaluation

NIH3T3 cells and MPS IVA mouse fibroblasts were transduced with IDLV carrying Cas9 with two sgRNA. Cells were collected 48 h later. Post-transduction to isolate genomic DNA (gDNA) using the Monarch Genomic DNA Purification Kit (New England Biolabs; Ipswich, MA, USA). High-fidelity PCR was performed using the gDNA with primers flanking the predicted target site on the *Galns* locus, Forward: 5′-TCACGCACCTCCTTGGC-3′ and Reverse: 5′-CCCTGAACACCTACCCCCA-3′. T7 endonuclease (EnGen Mutation Detection Kit, New England Biolabs, MA, USA) was used to digest the PCR products for mismatches following the manufacturer’s instructions. The digested PCR products were run on a 2% *w*/*v* agarose gel (ThermoFisher Scientific, Waltham, MA, USA) and visualized with the GelDoc XR+ system (Biorad; Hercules, CA, USA). GelAnalyzer 19.1 software was used to calculate the percentage of indels.

### 4.5. GALNS Enzyme Activity Assay

The intra- and extracellular GALNS enzyme activity in NIH3T3 and MPS IVA mouse fibroblasts was determined as previously described [20,43,47,68]. The GALNS activity was measured using 4-methylumbelliferyl-β-galactopyranoside-6-sulfate (4-MU-Gal-6S) (Toronto Chemicals Research, Vaughan, Ontario, Canada). The cell lysates collected on days 15 or 30 post-transduction were homogenized via sonication for three cycles of 10 s with 10% amplitude in a 25 mM Tris–HCl, pH 7.2, 1 mM PMSF buffer. The lysates were centrifuged for 10 min at 4 °C. The supernatant was collected in a new tube and incubated with 22 mM 4-MU-Gal-6S at 37 °C for 16 h. After incubation, 2 μL of β-galactosidase (10 mg/mL; Sigma-Aldrich) was added to the reaction and incubated for 1 h at 37 °C. The enzyme reaction was stopped by 968 μL of glycine-carbonate buffer (pH 10.5). The plate was read using exc/emi: 336/450 nm in a FLUOstart Omega microplate reader (BMG LabTech, Ortenberg, Germany). Media enzyme activity was conducted similarly. The enzyme activity (U) was defined as the amount of enzyme required to hydrolyze 1 nmol of substrate per hour, expressed either per milliliter (U/mL) for media or per milligram of protein (U/mg), based on a 4-methylumbelliferone standard curve (Sigma-Aldrich; St. Louis, MO, USA). The protein concentration was quantified using a BCA Protein Assay Kit (ThermoFisher Scientific, Waltham, MA, USA).

### 4.6. Mono-Sulfated KS Assay

The mono-sulfated KS levels in transduced NIH3T3 and MPS IVA mouse fibroblasts were determined by enzymatic digestion of KS polymers via liquid chromatography-tandem mass spectrometry (LC-MS/MS) as described previously [43,71]. Cell lysates were homogenized as described in 4.6. Extracted protein was treated with a cocktail of 5 μg/mL chondrosine as the internal standard (IS) and 1 mU keratanase II, with 50 nM 50 nM Tris-HCl (pH 7.0) in a 96-well Omega 10 K molecular weight cutoff filter plate (Pall 544 Corporation, Port Washington, NY, USA). This filter plate was positioned on a 96-well plate (ThermoFisher Scientific, Waltham, MA, USA) and incubated for 16 h. at 37 °C. After incubation, the plates were centrifuged at 2500× *g* for 15 min. The resulting disaccharides were isolated using a Hypercarb column (2.0 mm inner diameter, 50 mm length, 5 μM particle size; ThermoFisher Scientific, Waltham, MA, USA) at 60 °C. The binary gradient elution of 5 nM ammonium acetate (pH 11.0) was used, transitioning to 100% acetonitrile. The LC-MS/MS (6460, Agilent Technologies; Santa Clara, CA, USA) was operated in negative ion mode with electrospray ionization at a flow rate of 0.7 mL/min and a runtime of 10 min. MassHunter quantitative software B.03.01 was used to quantify the disaccharides using *m*/*z* ratios, with precursor and product ions for IS (354.3→193.1) and mono-sulfated KS (462→97), respectively. Final concentrations were analyzed as ng/mg.

### 4.7. Lysosomal Load Evaluation

The 30-day post-transduced cells were evaluated for lysosomal mass accumulation via LysoTracker Deep Red (Thermo Fischer Scientific #L12492, Waltham, MA, USA) staining [20,68]. Briefly, cells were stained with 50 nM LysoTracker in DMEM supplemented with 20% FBS for 60 min. After incubation, the cells were detached by trypsinization, washed twice with 1 × PBS, and resuspended in 1 × HBSS and Propidium Iodide (1: 1000, 1 mg/mL, Sigma-Aldrich #P4864, St. Louis, MO, USA), excluding non-viable cells. Exactly 50,000 single, live fibroblast events were acquired on a NovoCyte 3000 flow cytometer (Agilent Technologies, Santa Clara, CA, USA) using the 647 nm laser and 668 nm emission channel, and the data were processed in FlowJo^TM^ v10.10.0 software.

For microscopic assessment of lysosomal burden, 30-day-treated fibroblasts were seeded on glass coverslips precoated with 50 µg/mL poly-D-lysine. Cultures were incubated with 75 nM LysoTracker Deep Red for 1 h, rinsed twice in PBS, and fixed for 15 min at room temperature (RT) in 4% paraformaldehyde (Invitrogen, Waltham, MA, USA). The coverslips were mounted in ProLong^TM^ Glass Antifade Mountant with NucBlue (Invitrogen, Waltham, MA, USA), imaged using an AXIO Observer Z1 inverted fluorescent microscope (Carl Zeiss Microscopy, Oberkochen, Germany), and the images were quantified with ImageJ v1.53 software.

### 4.8. MPS IVA Mouse Model Maintenance

MPS IVA knock-out (KO) mice (*Galns*^-/-^, MKC) were developed on a C57BL/6 background and characterized previously [72]. This mouse has a deletion of 1.8 kb from part of intron 1 and exon 2, resulting in no functional GALNS activity. Three homozygous MKC male mice at birth were injected into the superficial temporal vein with different doses (10^12^, 10^11^, and 10^10^ TU/kg) to determine the optimum dose leading to the neonate’s survival. The optimal dose was used to inject the dual IDLV vectors with a single injection into 5 neonates in each experimental group. The controls were unaffected WT C57BL/6 mice. A total of 50 μL suspension was injected into each experimental group of neonates. The injected and control mice were weaned for 21 days and raised independently. Plasma was collected biweekly from mice of each experimental group, starting at 4 weeks of age, with body weight measurements taken weekly. The mice were euthanized at 16 weeks old with CO_2_ exposure. All animals were cared for, and experiments were conducted as approved by the Institutional Animal Care and Use Committee of Nemours Children’s Health under Protocol RSP21—12482-002.

### 4.9. Blood and Tissue Collection

Beginning 4 weeks post-injection, blood was drawn bi-weekly from the superficial temporal (facial) vein using a 22 G BD Eclipse Vacutainer needle (Fischer Scientific, Pittsburgh, PA, USA) and collected into EDTA-lined BD Microtainer tubes (BD Microtainer, Franklin Lakes, NJ, USA). At 16 weeks, blood was collected in EDTA and non-anticoagulant tubes (BD Microtainer, Franklin Lakes, NJ, USA). After centrifugation at 4 °C, the plasma was stored at −20 °C, whereas the serum was snap-frozen and kept at −80 °C until analysis.

During necropsy, mice were perfused transcardially with 0.9% (*w*/*v*) NaCl delivered through a Masterflex L/S Easy-Load II peristaltic pump (Sigma-Aldrich, St. Louis, MO, USA) via a 27 G × ¾ scalp-vein set (Fischer Scientific, Pittsburgh, PA, USA) inserted into the left ventricle. Following perfusion, the arms, brain, eyes, gonads, heart, kidneys, limbs, liver, lungs, skeletal muscle, spine, spleen, and trachea were harvested and either flash-frozen at −80 °C or fixed in 4% paraformaldehyde, according to downstream requirements.

## 5. GALNS Enzyme Activity Assessment for Plasma and Tissues

The measurement was performed as described in 4.6. Plasma was evaluated directly, but for tissues, 50 mg of each tissue was weighed and homogenized in Tris buffer (25 mM, 1 mM PMSF, pH 7.2) using OMNI homogenizing tubes in the Bead Mill Homogenizer (OMNI International, Kennesaw, GA, USA). The supernatant after centrifugation for 30 min at 4 °C was utilized for GALNS enzyme activity.

### 5.1. Toluidine Blue Staining

The hearts and knee joints, initially fixed in 10% formalin post-autopsy, were processed using the JB-4 resin method (JB-4 Plus Embedding Kit, EMS, Hatfield, PA, USA). The knee joints were subjected to decalcification with buffer containing 10% EDTA in 0.1 M Tris-HCl buffer (pH 7.0). The process was conducted over three weeks, with buffer changes made twice a week. The remaining steps were similar for both the heart and knee joints. The tissues were dehydrated overnight at RT through ascending ethanol solutions (25, 50, 75, 95, and 100%). After dehydration, the tissues were treated with infiltration using JB-4 Solution A and benzoyl peroxide solution overnight on a slow rotator. After infiltration, the samples were embedded in the correct orientation using JB-4 Solution B and the infiltration solution, which was mixed overnight in a vacuum desiccator. 0.5-μm-thick sections were cut with the Leica UC7 ultramicrotome instrument (Leica Biosystems, Deer Park, IL, USA) and stained with toluidine blue for further analysis. A double-blind analysis method was used for both tissues to score vacuolization and column structures on a scale of 0 (best) to 3 (worst).

### 5.2. Micro Computational Tomography (μCT)

Micro-CT scans were performed for the femur of all experimental groups using the SkyScan 1276 μCT System (Bruker, Manning Park, MA, USA). The femur was originally collected in 100% ethanol post-autopsy at 16 weeks. Before scanning, they were wrapped in salinated (0.9% saline) gauze to maintain hydration during the scan. The scanning was performed at an 8.0 μm pixel size, with an exposure of 1600 ms, a voltage of 85 keV, a current of 47 μA, and 512 projections. Three-dimensional volumetric models were constructed for the cortical and trabecular regions of interest. Bone morphometric parameters of each area of interest were evaluated using CTan 1.17.7.2 software, which quantified bone mineral density, bone and tissue volume, and cortical thickness.

### 5.3. Anti-GALNS Antibody Assessment

Plasma harvested from treated animals was screened for antibodies against GALNS using the procedure previously described [20,47]. Briefly, 96-well Falcon plates were coated overnight at 4 °C (humid chamber) with 100 µL of elosulfase alfa (Vimizim^®^, BioMarin, Novato, CA, USA) at 2 µg/mL prepared in 15 mM Na_2_CO_3_/35 mM NaHCO_3_/0.02% NaN_3_, pH 9.6. Plates were washed three times with 250 µL TBS-T (10 mM Tris-HCl, pH 7.5; 150 mM NaCl; 0.05% Tween-20) and blocked for 1 h at RT with 200 µL of 3% BSA in PBS (pH 7.2). After another TBS-T wash, duplicate plasma samples diluted 1: 100 in TBS-T were added with monoclonal anti-GALNS antibodies (Custom-made clone 2F5F2, Creative Biolabs, Shirley, NY, USA) and incubated for 2.5 h at 37 °C. Plates were washed four times with TBS-T, followed by 100 µL of HRP-conjugated goat anti-mouse IgG (1:1000 in TBS-T, Invitrogen #656120, Waltham, MA, USA) for 1 h at RT. After three washes with TBS-T and two with TBS, 100 µL ABTS substrate (Invitrogen #002024, Waltham, MA, USA) was applied for 30 min (RT). The reaction was terminated with 100 µL 1% SDS, and absorbance was read at 410 nm on a FLUOstar Omega microplate reader (BMG LabTech, Cary, NC, USA). Optical density (OD_410_) values were recorded; plasma from MKC mice receiving elosulfase alfa enzyme replacement therapy served as the positive control.

### 5.4. Anti-Cas9 Antibody Assessment

Anti-Cas9 antibody levels were quantified following previously described methods [20]. Falcon 96-well plates were coated overnight at 4 °C with 100 µL purified Cas9 (Cas9, Millipore, Burlington, MA, USA) at 100 µg/mL in the same carbonate buffer used above (15 mM Na_2_CO_3_/35 mM NaHCO_3_/0.02% NaN_3_, pH 9.6). Plates were washed three times with TBS-T and blocked for 1 h at RT with 3% BSA in PBS. After washing, 100 µL of mouse plasma (1: 100 in TBS-T) or serial dilutions of the anti-Cas9 monoclonal antibody sc-517386 were added in duplicate and incubated as described for the GALNS assay. Detection, washing, color development, and OD reading followed the abovementioned steps. Antibody concentrations (ng/mL) were determined by interpolating sample OD_410_ values against the anti-hCas9 standard curve.

### 5.5. IDLV-Related Toxicity

The use of dual IDLV carrying the respective expression cassette and Cas9; gRNA could lead to toxicity, which was determined by using alanine transaminase (EALT-100, BioAssay Systems, Hayward CA, USA) and aspartate transaminase (EASTR-100, BioAssay Systems, Hayward, CA, USA) according to the manufacturer’s instructions. The reactions were performed in 96-well plates, and the fluorescence was read using the FLUOstar Omega microplate reader (LabTech, Cary, NC, USA) at the wavelength specified by the supplier.

### 5.6. Statistical Analysis

Quantitative variables were analyzed in GraphPad Prism 9.5.0 (GraphPad Software, San Diego, CA, USA). Data were first screened for normality with the Shapiro-Wilk and Kolmogorov-Smirnov tests and for homogeneity of variances with Levene’s test. Variables that satisfied these assumptions are reported as mean ± standard error (SE) and compared with a two-tailed Student’s *t*-test (two groups) or one-way ANOVA followed by a post hoc Tukey’s Honest Significant Difference test (three or more groups). Variables that failed either assumption are expressed as the median with the 95% confidence interval and analyzed using the Mann–Whitney U test (two groups) or the Kruskal–Wallis test coupled with Dunn’s multiple-comparison procedure (three or more groups). Pre-specified contrasts included treatment versus WT, treatment versus untreated disease control, and inter-treatment comparisons. A *p*-value ≤ 0.05 was considered statistically significant. Biological replication consisted of five mice per treatment arm for in-vivo studies (*n* = 5) and three independent cultures per condition for in-vitro assays (*n* = 3). Given the proof-of-concept nature of this study, the sample sizes were limited to *n* = 3–5 per group. The authors acknowledge that this may limit the statistical power to detect more subtle therapeutic effects and that any trends not reaching statistical significance should be interpreted with caution as they may warrant further investigation in larger cohorts.

## 6. Conclusions

This proof-of-concept study provides compelling evidence for the therapeutic utility of IDLVs as a delivery vehicle for CRISPR/Cas9-mediated gene therapy for MPS IVA. The dual mechanism of episomal and HDR-driven *GALNS* expression, coupled with a good safety profile, yields significant and sustained biochemical correction in vitro and notable, albeit partial, therapeutic effects in vivo. These findings highlight the potential of this approach to address some of the limitations of current MPS IVA treatments.

The path to clinical translation will necessitate significant advancements in IDLV vector engineering to enhance episomal stability and HDR efficiency, optimize dosing and delivery strategies to improve in vivo outcomes and rigorous preclinical validation, including long-term safety and efficacy studies. The insights gained, combined with the rapid evolution of gene editing technologies and LV design, point to a promising trajectory for developing a more effective and potentially curative treatment for the devastating multi-systemic consequences of MPS IVA. Continued research focusing on these identified IDLV-specific areas of improvement is crucial for realizing the full therapeutic potential of this platform for MPS IVA and other LSDs.

## Data Availability

All available data are published.

## Supplementary Materials

The following supporting information can be downloaded at: https://www.mdpi.com/article/10.3390/ijms26146616/s1.

## Abbreviations

The following abbreviations are used in this manuscript:

MPS IVA	Mucopolysaccharidosis type IVA
LSD	Lysosomal Storage Disorder
GALNS	N-acetyl-galactosamine-6-sulfate sulfatase
GAGs	Glycosaminoglycans
KS	Keratan Sulfate
C6S	Chondroitin-6-sulfate
IDLV	Integrase-Deficient Lentiviral vector
CRISPR/Cas9	Clustered Regularly Interspaced Short Palindromic Repeats/CRISPR associated protein 9
HDR	Homology-Directed Repair
sgRNA	Single Guide RNA
ERT	Enzyme Replacement Therapy
HSCT	Hematopoietic Stem Cell Transplantation
GVHD	Graft-Versus-Host Disease
MOI	Multiplicity of Infection
EGFP	Enhanced Green Fluorescent Protein
WT	Wild-Type
