# Supplementary material for: Integrase-Deficient Lentiviral Vector as a Platform for Efficient CRISPR/Cas9-Mediated Gene Editing for Mucopolysaccharidosis IVA
Source: Int J Mol Sci. 2025 Jul 10;26(14):6616. doi: 10.3390/ijms26146616 (PMC12295186; doi:10.3390/ijms26146616)
Supplement: Supplementary file 1 [file ijms-26-06616-s001.zip › ijms-3714517-supplementary.pdf]

Supplemental Information

Integrase-Deficient Lentiviral Vector as a Platform for Efficient CRISPR/Cas9-mediated Gene Editing for Morquio A Syndrome

Fnu Nidhi [1,2] and Shunji Tomatsu [1,3,4*]

1 Nemours Children's Health, Wilmington, DE 19803, USA
2 Faculty of Arts and Sciences, University of Delaware, Newark, DE 19716, USA
3 Department of Pediatrics, Graduate School of Medicine, Gifu University, Gifu, Japan 10
4 Department of Pediatrics, Thomas Jefferson University, Philadelphia, PA, USA 11
* Correspondence: shunji.tomatsu@nemours.com (S.T.); +1-302-298-7336 (S.T.); Fax: +1-302-651-6888 (S.T.)

Supplementary Materials and Methods

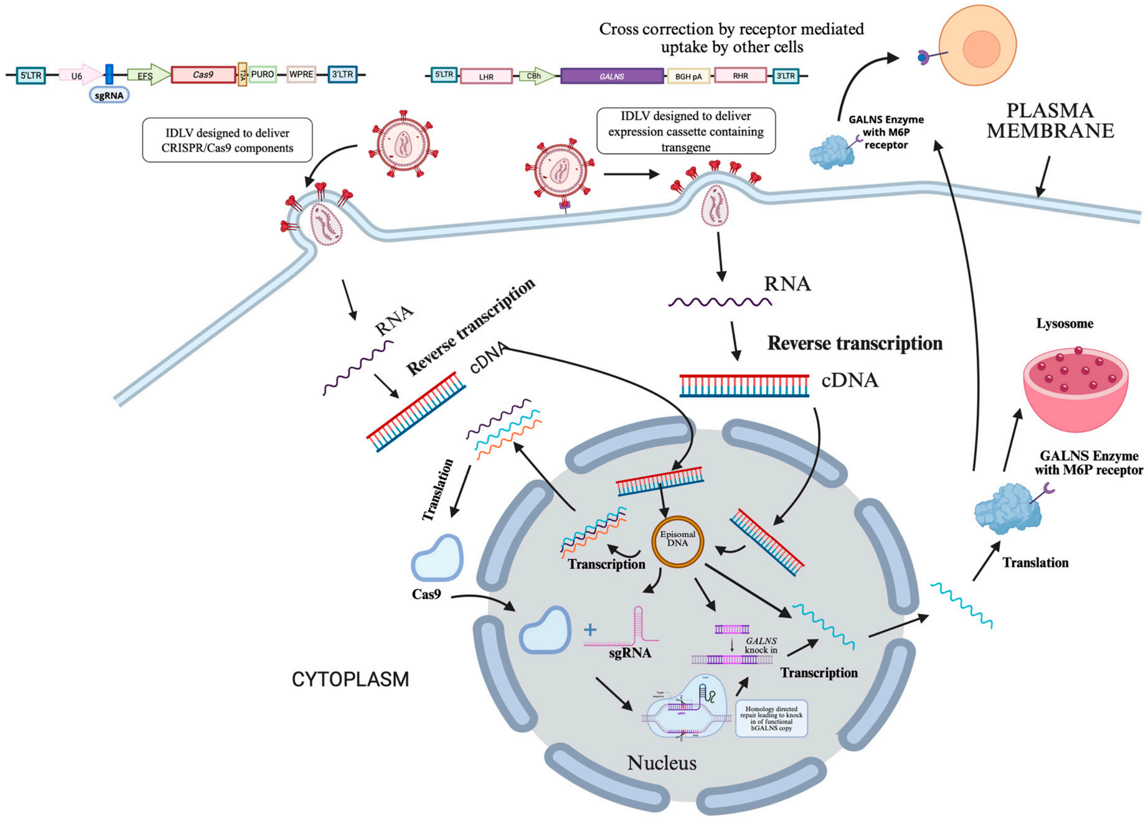

Supplementary Figure S1. IDLV-mediated CRISPR/Cas9 gene editing targeting strategy. Molecular mechanism of dual IDLV-mediated CRISPR/Cas9 delivery and homology-directed repair (HDR). IDLV particles encapsulate vectors encoding either CRISPR/Cas9 components or the donor template. Upon cellular entry, reverse transcription generates episomal DNA that transiently expresses Cas9 and sgRNA, facilitating targeted genomic double-stranded breaks (DSBs). Concurrently, episomal donor DNA with homology arms directs genomic integration through HDR. Episomal vector persistence provides an additional source of GALNS expression, independent of integration events. Cross-correction occurs through mannose-6-phosphate (M6P) receptor-mediated uptake of secreted GALNS enzyme by neighboring cells.

A. IDLV-mediated gene editing restores* GALNS *activity in MPS IVA mouse fibroblasts.

2.4 Vector Copy Number Determination

Briefly, cells were collected, and gDNA was isolated using a commercial genomic DNA purification kit (e.g., DNeasy Blood & Tissue Kit, Qiagen) following the manufacturer's protocol from 30-day transduced cells. This typically involved cell lysis, followed by proteinase K digestion to degrade proteins, and then RNase A treatment to remove RNA. Purified gDNA was subsequently resuspended in an appropriate buffer (e.g., Tris-EDTA buffer), and its concentration and purity were assessed by spectrophotometry (e.g., NanoDrop). Quantification of VCN was performed by digital droplet PCR (ddPCR) using a suitable ddPCR system (e.g., QX200 Droplet Digital PCR System, Bio-Rad). The ddPCR reactions were designed to quantify

the copy number of the integrated IDLV vector relative to a known mouse reference gene that exists at a stable copy number per diploid genome (e.g., Tfrc or Gapdh, typically 2 copies). Primers and fluorescently labeled probes (e.g., TaqMan probes) were specifically designed to amplify a unique sequence within the IDLV vector backbone (e.g., the psi (Ψ) packaging signal or another vector-specific region) and an area of the chosen mouse reference gene [1].

Supplemental Figure S2: Vector copy number in transduced MPS IVA mouse fibroblasts.

Vector copy number (VCN) per diploid genome was determined by ddPCR in MPS IVA mouse fibroblasts 30 days following transduction with IDLV:GALNS (donor-only), IDLV:gRNA1/GALNS, or IDLV:gRNA2/GALNS. Untreated (UT) fibroblasts served as a negative control. Data are presented as the mean ± SD for three biological replicates (n = 3), with individual data points overlaid. Statistical significance was assessed by one-way ANOVA followed by Tukey's multiple comparisons test. Significant differences are indicated as ** $p < 0.01$, **** $p < 0.0001$. The analysis demonstrates significantly higher VCN in cells transduced with HDR-competent vectors (IDLV:gRNA1/GALNS and IDLV:gRNA2/GALNS) compared to the donor-only vector, with IDLV:gRNA1/GALNS achieving the highest integration.

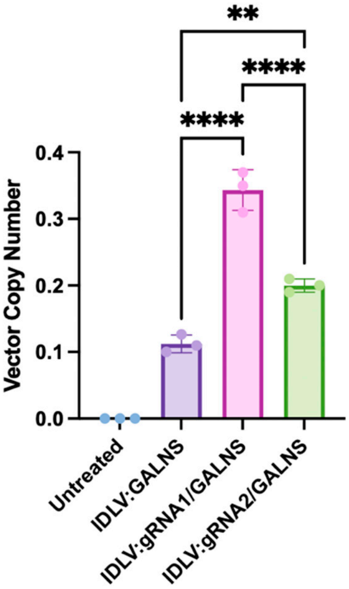

B. In vivo validation of the IDLV-mediated CRISPR/Cas9 gene editing strategy

2.6 Determination of Optimal In Vivo Vector Dose and Neonatal Survival Study

To determine a safe and effective dose for in vivo administration, a dose-escalation study was performed in newborn MPS IVA mice. Neonatal (P1-P3) MPS IVA mice were randomly assigned to groups and administered with IDLV:GALNS with IDLV:gRNA1/GALNS via facial vein injections at varying doses: 5, 2.5, 1.25, 1 × 10^{10} TU/kg, 10^{11} TU/kg10^{12} TU/kg. A control group received a vehicle (saline). Mice were monitored daily for survival until the endpoint at 3 weeks.

Supplemental Figure S3: Dose-dependent survival of newborn MPS IVA mice following IDLV:CRISPR/Cas9 combined administration.

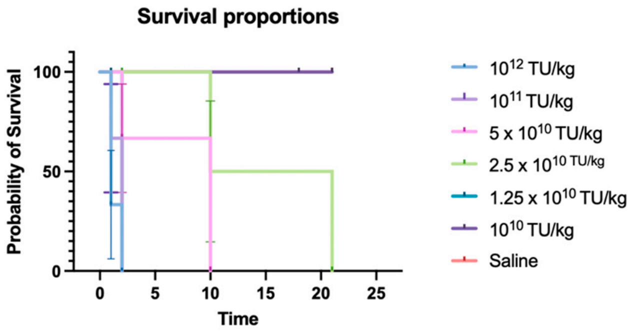

Kaplan-Meier survival curves for newborn MPS IVA mice treated with increasing doses of vector, with each dose group, including the saline control, starting with a total of n = 3 mice. (1 × 10^{12} TU/kg, n = 1 (survived longer then initial n = 3); 1 × 10^{11} TU/kg, n = 1; 5 × 10^{10} TU/kg, n = 1; 2.5 × 10^{10} TU/kg, n = 1; 1.25 × 10^{10} TU/kg, n = 2; 1 × 10^{10} TU/kg, n = 3) or saline (control, n = 3). Mice alive at 21 days were censored for this analysis and utilized for subsequent experiments. The results demonstrate a dose-dependent toxicity: doses of 1 × 10^{11}

TU/kg and higher led to acute mortality within the first few days. The 5 × 10^{10} TU/kg dose resulted in 0% survival by days 10-12, and the 2.5 × 10^{10} TU/kg dose showed 0% survival by days 21-22. In contrast, doses of 1.25 × 10^{10} TU/kg and 1 × 10^{10} TU/kg, along with saline, showed 100% survival throughout the 3-week observation period. Statistical significance between survival curves was determined using the Log-rank (Mantel-Cox) test. This study identified 1 × 10^{10} TU/kg as the optimal dose for subsequent experiments based on survival and tolerability within the initial 3-week period.

2.6 Body Weight Analysis

The body weight of MPS IVA mice was monitored biweekly following neonatal vector administration. Mice from Wild-Type (WT), Untreated MPS IVA (Untreated), IDLV-GALNS (donor-only), IDLV:gRNA2/GALNS, and IDLV:gRNA1/GALNS groups were weighed weekly. Average body weights for each group at each time point were calculated and plotted to observe growth trends. Statistical analysis, such as a two-way ANOVA with Tukey's or Sidak's multiple comparisons test, was performed to compare body weights between groups at each time point or across the study duration.

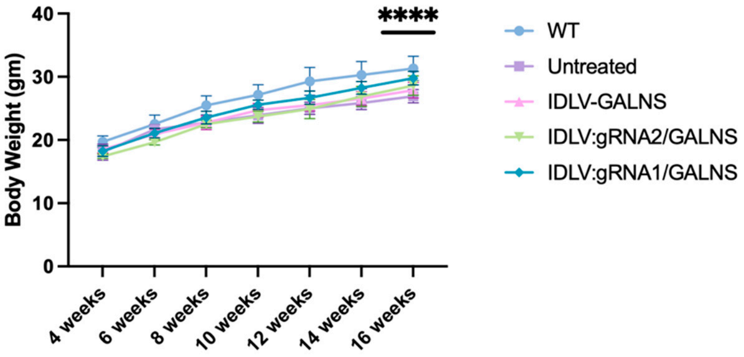

Supplementary Figure S4: Longitudinal body weight assessment of MPS IVA mice following neonatal gene therapy.

Body weight (grams) of Wild-Type (WT), Untreated MPS IVA, IDLV-GALNS, IDLV:gRNA2/GALNS, and IDLV:gRNA1/GALNS treated mice measured at 4, 6, 8, 10, 12, 14, and 16 weeks of age. Data are presented as mean ± SEM (Standard Error of the Mean). Statistical comparisons between groups were performed (e.g., using two-way ANOVA with post-hoc tests). Significant differences are indicated (e.g., ****p < 0.0001 comparing WT to Untreated at 16 weeks, or other relevant comparisons as per your statistical analysis).

2.8 Systemic IDLV delivery (10^{10} TU/kg) mitigates cardiac storage but yields only focal skeletal benefit.

Supplementary Table S1: Heart pathology scores

Tissue	WT	UT	IDLV:GALNS	IDLV:gRNA1:GALNS	IDLV:gRNA2:GALNS
**Mitral valve**	0.00±0.00	3.00±0.00	1.86±1.03	1.90±0.89	0.63±1.25
**Mitral Valve Base**	0.00±0.00	2.88±0.25	1.86±1.03	1.30±0.97	0.67±1.15
**Heart Muscle**	0.00±0.00	2.88±0.25	1.64±1.18	1.70±0.97	1.33±0.58

Supplementary Table S2: Vacuolization pathology scores

Region	IDLV:GALNS	IDLV:gRNA1:GALNS	IDLV:gRNA2:GALNS	UT	WT
**Femur Articular Cartilage**	2.80 ± 0.27	2.30 ± 0.76	3.00 ± 0.00	2.90 ± 0.22	0.00 ± 0.00
**Femur Growth Plate**	2.80 ± 0.45	2.40 ± 0.65	2.80 ± 0.27	3.00 ± 0.00	0.00 ± 0.00
**Meniscus**	3.00 ± 0.00	2.50 ± 0.71	2.80 ± 0.27	2.90 ± 0.22	0.00 ± 0.00
**Tibia Articular Cartilage**	2.80 ± 0.45	2.40 ± 0.65	2.70 ± 0.27	3.00 ± 0.00	0.00 ± 0.00
**Tibia Growth Plate**	3.00 ± 0.00	2.50 ± 0.71	2.70 ± 0.27	3.00 ± 0.00	0.00 ± 0.00

Supplementary Table S2: Column structure pathology scores

Structure	IDLV:GALNS	IDLV:gRNA1:GALNS	IDLV:gRNA2:GALNS	UT	WT
**Femur Articular Cartilage**	2.50 ± 0.00	2.40 ± 0.58	2.60 ± 0.20	2.80 ± 0.24	0.00 ± 0.00
**Femur Growth Plate**	2.80 ± 0.24	2.30 ± 0.51	2.60 ± 0.20	3.00 ± 0.00	0.00 ± 0.00
**Tibia Articular Cartilage**	2.80 ± 0.24	2.30 ± 0.51	2.70 ± 0.24	2.80 ± 0.24	0.00 ± 0.00
**Tibia Growth Plate**	2.90 ± 0.20	2.30 ± 0.51	2.60 ± 0.20	2.80 ± 0.24	0.00 ± 0.00

References:

1. Celik, B., Rintz, E., Sansanwal, N., Khan, S., Bigger, B., and Tomatsu, S. (2024). Lentiviral Vector-Mediated *Ex Vivo* Hematopoietic Stem Cell Gene Therapy for Mucopolysaccharidosis IVA Murine Model. Hum Gene Ther *35*, 917–937. 10.1089/hum.2024.094.
